# Integrated Analysis of the Transcriptome and Metabolome Reveals Genes Involved in Terpenoid and Flavonoid Biosynthesis in the Loblolly Pine (*Pinus taeda* L.)

**DOI:** 10.3389/fpls.2021.729161

**Published:** 2021-10-01

**Authors:** Jipeng Mao, Linwang Huang, Manyu Chen, Weishan Zeng, Zhiheng Feng, Shaowei Huang, Tianyi Liu

**Affiliations:** ^1^Guangdong Key Laboratory for Innovative Development and Utilization of Forest Plant Germplasm, College of Forestry and Landscape Architecture, South China Agricultural University, Guangzhou, China; ^2^Taishan Hongling Seed Orchart, Jiangmen, China; ^3^State Key Laboratory for Conservation and Utilization of Subtropical Agro-Bioresources, South China Agricultural University, Guangzhou, China

**Keywords:** *Pinus taeda*, transcriptome, metabolome, flavonoids, terpenoids

## Abstract

Loblolly pine (*Pinus taeda* L.) is an important tree for afforestation with substantial economic and ecological value. Many metabolites with pharmacological activities are present in the tissues of *P. taeda*. However, the biosynthesis regulatory mechanisms of these metabolites are poorly understood. In the present study, transcriptome and metabolome analyses were performed on five tissues of *P. taeda*. A total of 40.4 million clean reads were obtained and assembled into 108,663 unigenes. These were compared with five databases, revealing 39,576 annotated unigenes. A total of 13,491 differentially expressed genes (DEGs) were observed in 10 comparison groups. Of these, 487 unigenes exhibited significantly different expressions in specific tissues of *P. taeda*. The DEGs were explored using Gene Ontology and Kyoto Encyclopedia of Genes and Genomes metabolic pathway analysis. We identified 343 and 173 candidate unigenes related to the biosynthesis of terpenoids and flavonoids, respectively. These included 62 *R2R3-MYB*, 30 *MYB*, 15 *WRKY*, seven *bHLH*, seven *ERF*, six *ZIP*, five *AP2*, and one *WD40* genes that acted as regulators in flavonoid and/or terpenoid biosynthesis. Additionally, metabolomics analysis detected 528 metabolites, among which 168 were flavonoids. A total of 493 differentially accumulated metabolites (DAMs) were obtained in 10 comparison groups. The 3,7-Di-O-methyl quercetin was differentially accumulated in all the comparison groups. The combined transcriptome and metabolome analyses revealed 219 DEGs that were significantly correlated with 45 DAMs. Our study provides valuable genomic and metabolome information for understanding *P. taeda* at the molecular level, providing a foundation for the further development of *P. taeda-*related pharmaceutical industry.

## Introduction

Pines are typical gymnosperms, and more than 80 pine species are distributed widely across the world. In addition to their use for lumber, pines also have ornamental and medicinal value (Lee et al., 2001; Kelkar et al., 2006; Olatunde et al., 2017). Needles are the main medicinal tissues of pines. Pine needle extracts are rich in amino acids, vitamins, carotenoids, phenols, unsaturated fatty acids, terpenoids, and water-soluble flavonoids. They possess significant healing and pharmacological properties. These include activities against cancer, cardiovascular, cerebrovascular, hypertension, hyperlipidemia, and Alzheimer's diseases (Jeon and Kim, 2006). Pine bark extracts are rich in natural antioxidant oligomeric proanthocyanidins (OPCs) and have anti-inflammatory (Shi et al., 2020; Vinueza et al., 2020), antiviral (Snehal et al., 2019), and antiaging properties (Yokozawa et al., 2011). Most of the pharmacological components of pine extracts are terpenoids and flavonoids, which have a great functional diversity in other plant species (Farhoudi and Lee, 2013; Li et al., 2018; Guo et al., 2020).

Terpenoids are one of the most important plant metabolites with a general formula of (C5H8)n. They are common in plants and often function in biological defense and developmental processes (Das et al., 2013; Hijaz et al., 2016). All terpenoids are derived from the common five-carbon structural unit precursor isopentenyl diphosphate (IPP) and its isomer dimethylallyl diphosphate (DMAPP). IPP/DMAPP can be generated *via* two independent and cooperative pathways, namely the mevalonate (MVA) and 2-C-methyl-D-erythritol-4-phosphate (MEP) pathways, which occur in the cytoplasm and plastids, respectively (Zenoni et al., 2010; Heuston et al., 2012). Flavonoids are synthesized through a complex process that originates from the phenylpropanoid pathway. L-Phenylalanine is the initial substrate, and it forms quercetin, catechin, epicatechin, anthocyanidin, and other flavonoids under the action of chalcone synthase (CHS), chalcone isomerase (CHI), flavanone 3-hydroxylase (F3H), dihydroflavanol 4-reductase (DFR), anthocyanidin synthase, and other phenylpropanoid pathway enzymes (Sharma and Dixon, 2005; Payyavula et al., 2013). The biosynthesis of these metabolites is regulated by gene actions that are poorly understood.

Transcriptome sequencing (RNA-Seq) technology can assist in the discovery and identification of genes involved in metabolite biosynthesis, especially for species without reference genomes. Many plant genes involved in the biosynthesis of terpenoids, flavonoids, and other pharmacologically active components have been successfully identified by RNA-Seq. Examples include the identification of flavonoid-related genes in *Ginkgo biloba* (Guo et al., 2020), *Apocynum venetum* (Gao et al., 2019), *Actinidia arguta* (Li et al., 2018), and *Capsicum annuum* (Liu et al., 2020). Terpenoid-related genes have been found in *Salvia officinalis* (Ali et al., 2017), *Chamaemelum nobile* L. (Liu et al., 2019), *Magnolia champaca* (Dhandapani et al., 2017), and phenolic acid-related genes in *Cyclocarya paliuru*s (Lin et al., 2020). Although some terpenoid-related genes have been identified in certain pines, flavonoid-related genes and other bioactive components have not been reported. Similar to transcriptomics, metabolomics is an important component of systems biology that can be used to identify and quantify endogenous small molecule metabolites (Kaiser, 2012). When combined with other “omics” tools, metabolomics can help elucidate gene functions, metabolite biosynthesis pathways, and regulatory mechanisms. For example, Xin et al. (2019) studied root growth regulation mechanisms in response to nitrogen availability in rice using the transcriptome and metabolome. Lang et al. (2019) determined the role of anthocyanin metabolism in *Michelia maudiae via* the transcriptome and metabolome.

*Pinus taeda* is widely distributed globally and is rich in germplasm resources. It is an important tree for afforestation and timber production worldwide. *P. taeda* was one of the first forest tree species used for genetic improvement. In this study, transcriptome and metabolome analyses were used to investigate the gene expression and metabolic differences in different tissues of *P. taeda* to provide insights into the genetic association of small-molecule metabolites. We studied the correlation between the gene transcription level and metabolite accumulation. Our results will enrich the plant omics database and provide useful information for the directed genetic improvement of *P. taeda* and its application in the pharmaceutical industry.

## Materials and Methods

### Plant Materials

Three 8-year-old *P. taeda* trees planted in the Yingde Research Institute of Forestry in Guangdong Province, China, with a similar growth state and free of pests and diseases were selected for sampling. The buds (SY), needles (SZ), twigs (ST), bark (SP), and roots (SG) were collected separately from each tree at the same time during the blooming period. The freshly collected materials were immediately frozen in liquid nitrogen and stored at −80°C until total RNA and metabolite extraction.

### RNA-Seq and Functional Annotation

Total RNA from each sample was extracted as previously described (Mao et al., 2019). The cDNA libraries were constructed following the protocol described by Foucart et al. (2006). Libraries were sequenced with 2 × 150 paired-end reads using the Illumina Novaseq6000 platform at the Science Corporation of Gene (Guangzhou, China). The Trinity method (Haas et al., 2013) was used to assemble high-quality reads into unigenes. The RNA sequence data were deposited in the National Center for Biotechnology Information (NCBI) Sequence Read Archive (SRA) under the study accession number SRP304195. Unigenes were then queried against The Uniprot, Non-redundant (Nr), Kyoto Encyclopedia of Genes and Genomes (KEGG), Eukaryotic Orthologous Groups of protein (KOG), and Gene Ontology (GO) public databases with an *E*-value threshold <10^−5^ to obtain functional annotations. The GO functional classifications and KEGG pathway analyses were conducted using the Web Gene Ontology Annotation Plot (WEGO) (Ye et al., 2006) and KEGG automatic annotation servers, respectively.

### Sample Preparation and Extraction

The samples were freeze-dried and crushed using a mixer mill (MM400, Retch) with zirconia beads for 2.0 min at 30 Hz. The powder (100 mg) was weighed and submerged in 0.7 ml of 70% aqueous methanol, and extracted for 12 h with constant shaking at 4°C. Following centrifugation at 10,000 *g* for 10 min, the supernatant was absorbed and filtrated (0.22-μm pore size) for ultra-performance liquid chromatography-tandem mass spectrometry (UPLC-MS/MS) analyses by Wuhan Metware Biotechnology Co., Ltd., China.

### UPLC–ESI–MS/MS Conditions

The sample extracts were analyzed based on the UPLC–ESI–MS/MS (electrospray ionization, ESI) system. The UPLC conditions were conducted as previously described (Zhao et al., 2020). Briefly, the UPLC column was a Water ACQUITY UPLC HSS T3 C18 (1.8 μm, 2.1 × 100 mm). The mobile phase consisted of solvent A, which was pure water with 0.04% acetic acid, and solvent B was acetonitrile with 0.04% acetic acid. The gradient program was 95% A and 5% B at 0 min, a linear gradient to 5% A and 95% B within 10 min, and 5% A and 95% B for 1 min, and 95% A and 5% B adjusted within 0.1 min and maintained for 2.9 min. The flow rate was 0.35 mL/min with a column temperature of 40°C, and an injection volume of 4 μl. The MS/MS conditions used an electrospray ionization temperature of 550°C, ion spray voltages of 5,500 V (positive ion mode) and −4,500 V (negative ion mode), ion source gas I (50 psi), gas II (60 psi), and curtain gas (30 psi), and the collision-activated dissociation was set to high. Each ion pair was scanned based on the optimized collision energy and declustering potential in the triple quadrupole (QQQ).

### Differential Expression and Quantitative Real-Time PCR Analyses

The expression levels of the unigenes were normalized and calculated as the value of fragments per kilobase of transcripts per million mapped fragments (FPKM). Differentially expressed genes (DEGs) identification was conducted using the R package DESeq (http://bioconductor.org/packages/release/bioc/html/DESeq.html), and the expression patterns were obtained *via* hierarchical clustering analysis (Wang et al., 2010). A total of 16 candidate genes were selected to investigate the expression profiles by qRT-PCR analysis. Reverse transcription was performed using a PrimeScript TMRT reagent kit with a gDNA Eraser (Code No: RR047A, TaKara, Dalian, China) following the instructions of the manufacturer. The primers for the 16 candidate gene sequences were designed using Primer Premier 5.0, and *PtActin* (F: GAGCAAAGAGATCACTGCACTTG; R: CTCATATTCGGTCTTGGCAATCC) was selected as an internal control. The qRT-PCR analyses were performed using the LightCycler 480 System (Roche, Basel, Switzerland) and a TB Green Premix Ex Taq II kit (Code No: RR820A, TaKara). The amplification conditions were as follows: one cycle of 95°C for 30 s, followed by 40 cycles of 95°C for 5 s, 55°C for 30 s, and 72°C for 30 s. Three biological replicates of each tissue of *P. taeda* were assessed, and each qRT-PCR reaction included three technical replicates. The relative expression levels of the candidate genes were calculated using the 2^−ΔΔCt^ method (Livak and Schmittgen, 2001).

### Qualitative and Quantitative Analysis of Metabolites

Qualitative and quantitative analyses of the metabolites were performed using secondary spectral information based on the public metabolite database and the self-built MWBD database (Wuhan Metware Biotechnology Co., Ltd., China). The characteristic ions of each substance were screened out by the multiple reaction monitoring of QQQ (Wei et al., 2013), and the signal strengths of the characteristic ions were obtained in the detector. The isotope signal and duplicate signals of the K^+^, Na^+^, and ${\text{NH}}_{4}^{+}$ ions were excluded during the analysis. The mass spectrometry file under the sample was opened with Analyst 1.6.3 software to carry out the integration and correction of chromatographic peaks, and the relative content of the corresponding substances in the peak area of each chromatographic peak was calculated. Finally, all the chromatographic peak area integral data were derived. To compare the contents of each metabolite in different samples, the mass spectrum peaks detected in each metabolite were calibrated in different samples, which were based on the retention time and peak pattern.

### Identification of Differentially Accumulated Metabolites and Statistical Analyses

Multivariate principal component analysis (PCA) and orthogonal partial least squares-discriminant analysis (OPLS-DA) were conducted using the base package and “MetaboAnalystR” in R. The multivariate analysis of variable importance in projection (VIP) in the OPLS-DA model was used to initially screen differentially accumulated metabolites (DAMs). The DAMs were identified based on a VIP ≥ 1 and fold-change ≥ 2 or ≤ 0.5. *K*-means analysis and heatmap analysis based on hierarchical clustering were performed in R. Functional annotation and enrichment analysis of the DAMs were conducted based on the KEGG database.

### Correlation Analysis of the Transcriptome and Metabolome Data

Based on the transcriptome and metabolome data, Pearson's correlation tests were used to explore the correlations between the DEGs and DAMs. Only the detected correlations with a Pearson's correlation coefficient (PCC) value ≥ 0.7 and *P* ≤ 0.05 were selected. A nine-quadrant graph was used to show the DEGs and DAMs in each group with a PCC ≥ 0.8 among different groups using “ggplot2” and “getopt” in R. In addition, the DEGs and DAMs were mapped to the KEGG pathway database to obtain their common pathway information.

## Results

### RNA-Seq and Assembly and Functional Annotation

**Figure 1:**
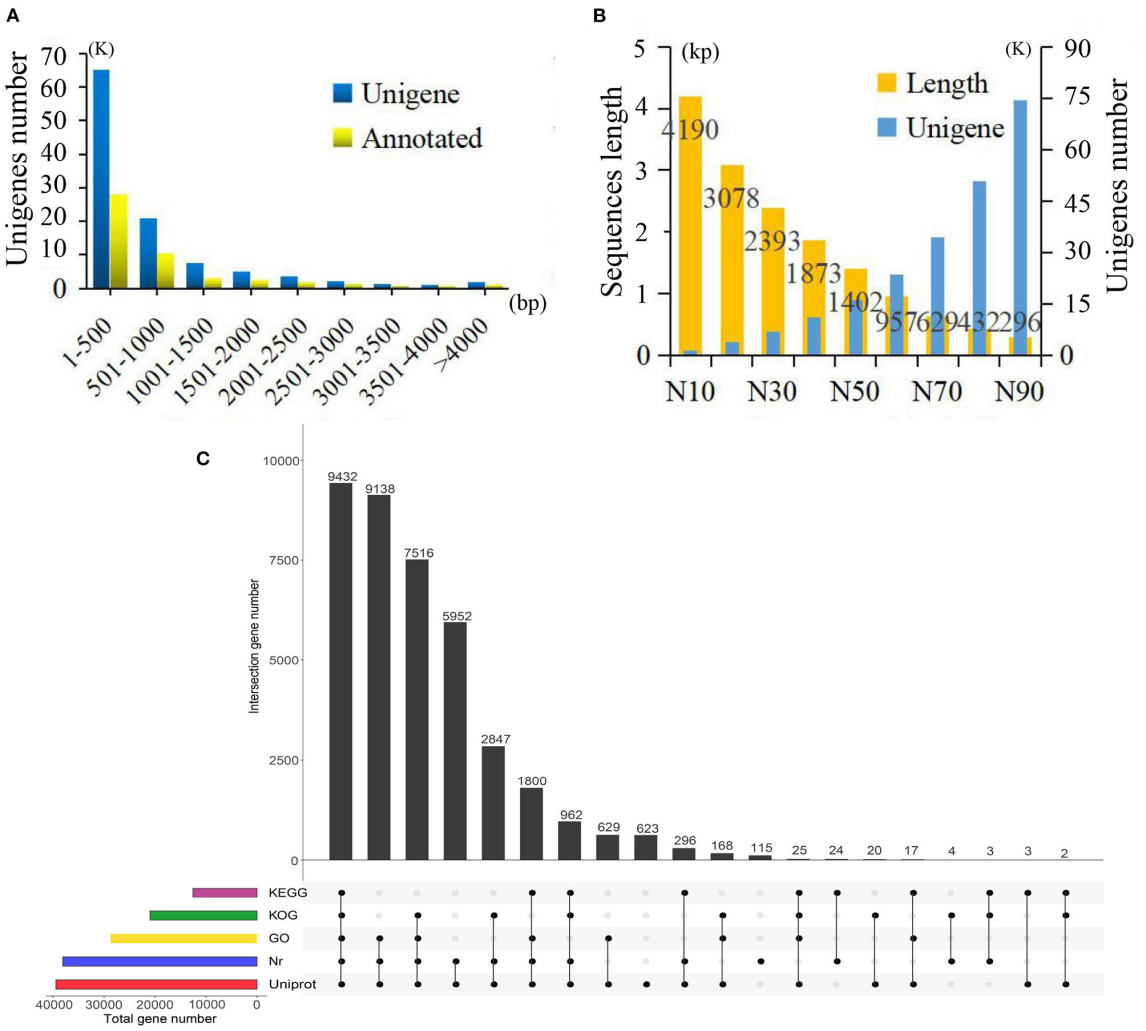
Summary of the assembly and annotations. **(A)** Sequence length distribution of unigenes and annotations. **(B)** Sequence length and number of unigenes under different assembly indices. **(C)** Annotations information obtained from five different databases.

In total, 15 cDNA libraries from the SY, SZ, ST, SP, and SG tissues of *P. taeda* were constructed and sequenced (Supplementary Table 1). The raw reads of the libraries were deposited in the NCBI Sequence Read Archive (SRA) database (accession number: SRP304195). After quality appraisal and low-quality data screening, 404,015,044 high-quality reads were obtained and assembled into 108,663 unigenes, with an average length of 776 bp, mean GC content of 41.54%, and N50 of 1,402 bp. Among these unigenes, the maximum length was 16,426 bp, the minimum length was 187 bp, and the length of 12,290 (11.31%) unigenes exceeded 1.5 kb (Figures 1A,B). A total of 39,576 (36.42%) unigenes were annotated by BLAST alignment using five public databases. In summary, 39,430 (36.28%), 38,089 (35.05%), 28,752 (26.43%), 20,979 (19.31%), and 12,564 (11.56%) unigenes were annotated in the UniProt, Nr, GO, KOG, and KEGG databases, respectively (Figure 1C).

Based on the Nr database, 14,282 (13.14%) unigenes exhibited significantly higher homology with sequences from *Picea sitchensis* than *P. taeda* and other species. Unigenes annotated in the GO database were mainly distributed in three categories with 15 GO terms. Within the categories of biological process, cellular component, and molecular function, the largest GO terms were “metabolic process,” “membrane part,” and “binding,” respectively (Supplementary Figure 1). Unigenes matched with the KEGG database were mainly included in the “global and overview maps,” “carbohydrate metabolism,” “translation,” and “signal transduction” pathways (Supplementary Figure 2).

### DEGs Identification and Enrichment Analyses

**Figure 2:**
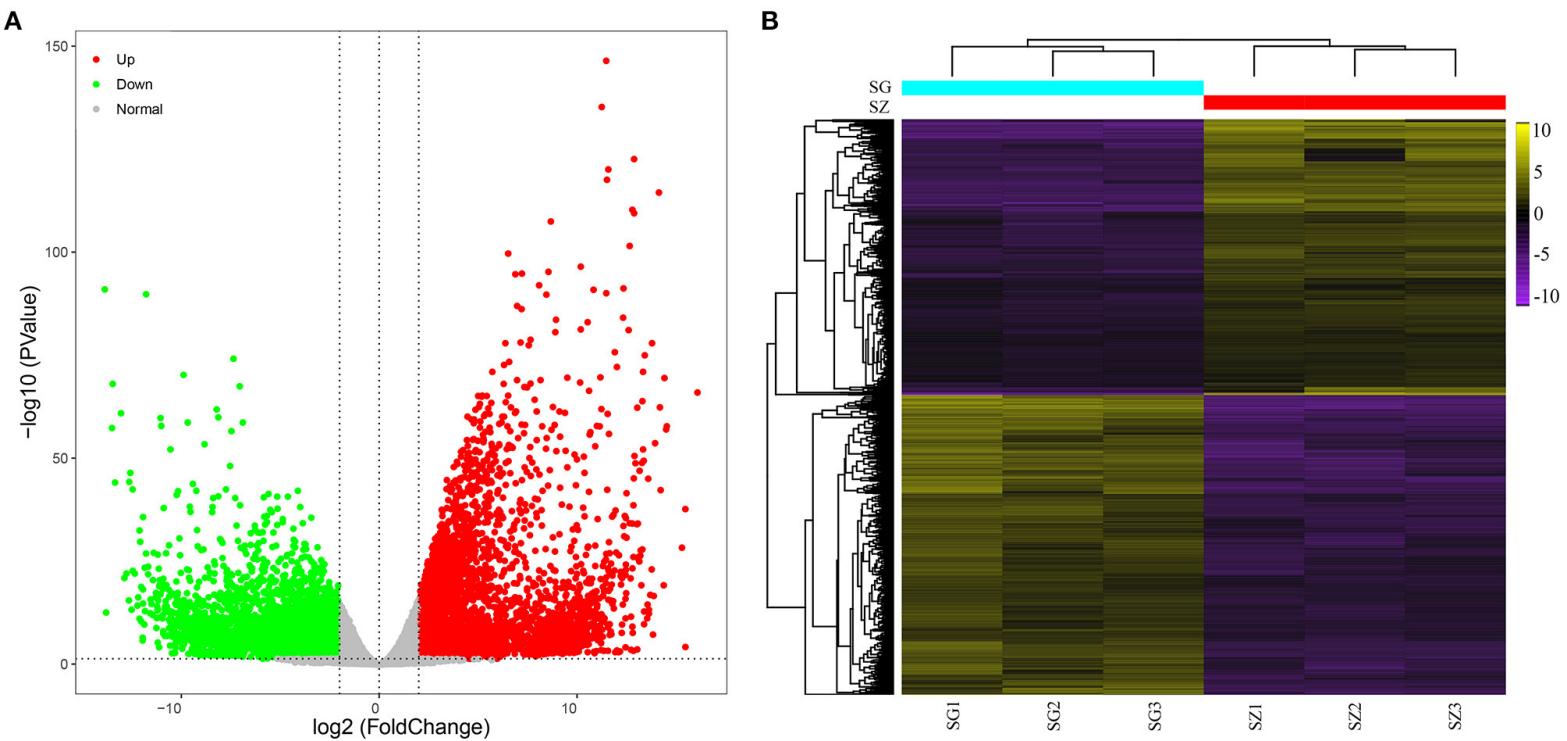
Analysis of differentially expressed genes. **(A)** Volcano plot of differentially expressed genes between SG and SZ libraries, red and green dots represent the significantly upregulated and downregulated genes, respectively. **(B)** Heat map of differentially expressed genes between SG and SZ libraries based on hierarchical clustering analysis.

The RPKM values were calculated for each unigene by setting |log_2_(fold change)| ≥ 2 and *P* ≤ 0.05 as thresholds for significant DEGs selection. A total of 13,491 significant DEGs were observed in 10 comparison groups (Table 1, Supplementary Table 2). Among these, the largest number of significant DEGs (3,516 upregulated and 4,623 downregulated genes) was detected between the SG and SZ libraries (Figure 2A).

**Table 1:** Summary of the DEGs and DAMs among 10 library pairs.

**Group**	**Up^a^**	**Down^a^**	**Total^a^**	**Up^b^**	**Down^b^**	**Total^b^**
SZ_vs_SP	3,567	3,553	7,120	155	147	302
SY_vs_SP	1,484	2,239	3,723	97	149	246
ST_vs_SG	884	1,283	2,167	26	193	219
SG_vs_SP	270	1,014	1,284	135	46	181
ST_vs_SP	271	1,577	1,848	57	138	195
SY_vs_SG	2,797	2,398	5,195	91	203	294
SY_vs_SZ	2,926	4,229	7,155	102	170	272
SG_vs_SZ	3,516	4,623	8,139	169	112	281
ST_vs_SZ	2,727	4,998	7,725	99	174	273
SY_vs_ST	1,939	1,150	3,089	106	83	189

a *genes;*

b *metabolites*.

The hierarchical clustering of the DEGs of the SG and SZ comparison groups is shown in Figure 2B. Yellow and purple indicate high and low expression levels, respectively. The expression patterns of the SG and SZ libraries revealed that SG_1, SG_2, and SG_3 and SZ_, SZ_2, and SZ_3 were classified into the same cluster.

**Figure 3:**
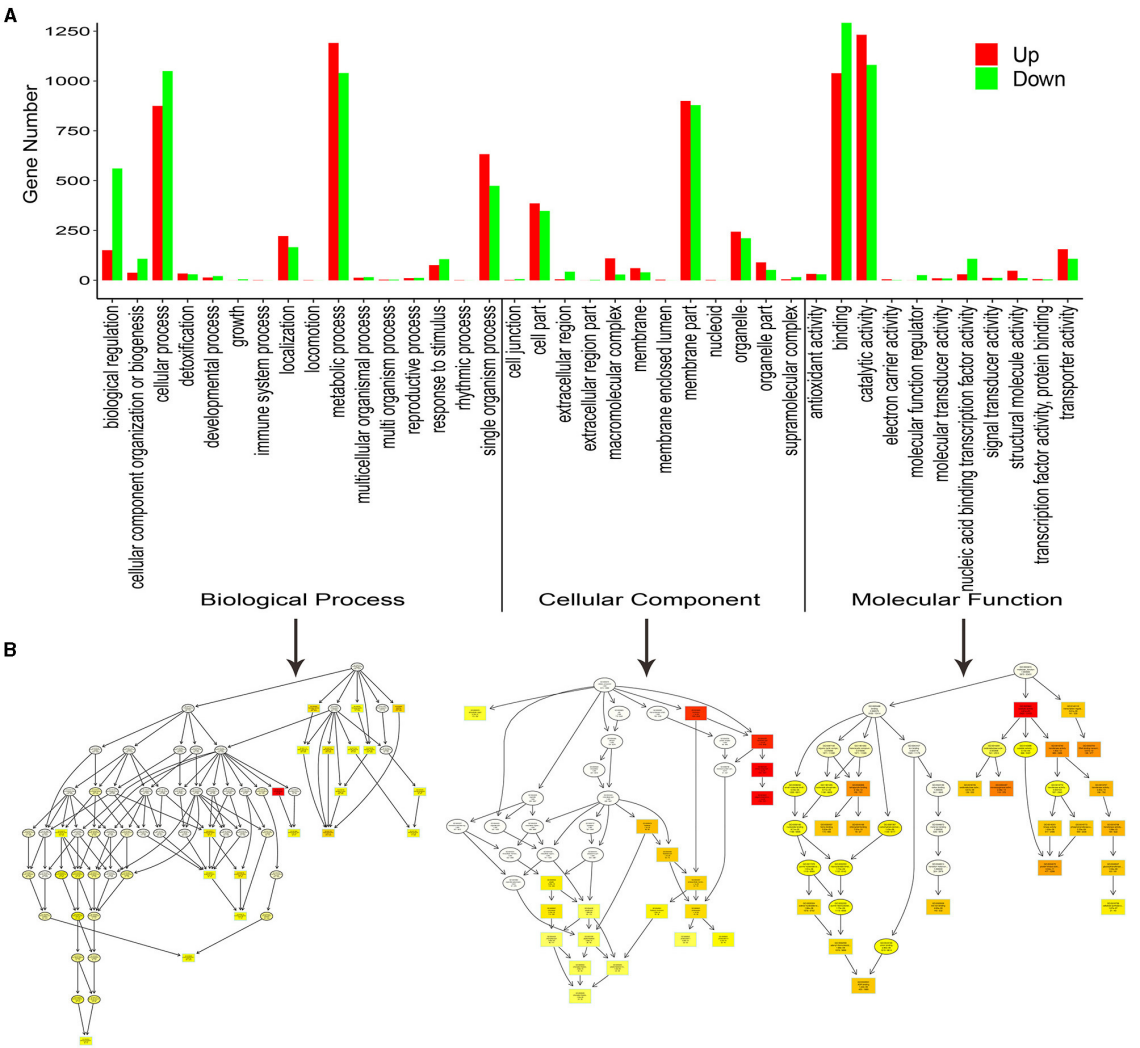
Functional GO and Kyoto Encyclopedia of Genes and Genomes (KEGG) pathway classification of differentially expressed genes. **(A)** Enriched GO terms are shown on the x-axis, and the numbers of the up- and down-regulated differentially expressed genes are shown on the y-axis. **(B)** Directed acyclic graphs of three main categories are displayed in thumbnail view. The nodes are color-based on the *q*-value, and a darker color indicates a higher confidence level. The GO terms are presented at the horizontal node position.

To better understand the biological functions of the DEGs, the significant DEGs from 10 comparison groups were functionally categorized using GO and KEGG enrichment analyses. For GO analysis, the significant DEGs between the SG and SZ libraries were mainly enriched in “cellular process,” “metabolic process,” “single organism process,” “cell part,” “membrane part,” “binding,” and “catalytic activity”(Figure 3A). The “metabolic process” subcategory was composed mainly of downregulated DEGs, whereas the “catalytic activity” subcategory was composed mainly of upregulated DEGs. Directed acyclic graphs were also used to illustrate the GO structure results (Figure 3B). Similar to the functional categories, three GO-directed acyclic graphs were constructed for biological process, cellular component, and molecular function, respectively. For the KEGG analysis, the significant DEGs between SG and SZ libraries were mainly enriched in the “metabolic pathways” and “biosynthesis of secondary metabolites” pathways (Supplementary Figure 3). The “metabolic pathways” and “biosynthesis of secondary metabolites” pathways were composed mainly of downregulated and upregulated DEGs, respectively. The significant DEGs in the other nine comparison groups showed similar GO and KEGG enrichment characteristics (Figures not shown).

### Identification of Unigenes Related to Terpenoid and Flavonoid Biosynthesis

In this study, 343 and 173 candidate unigenes related to flavonoid and terpenoid biosynthesis pathways, respectively, were identified from the *P. taeda* transcriptome. For flavonoid biosynthesis-related genes, 239, 80, and 24 unigenes were involved in the phenylpropanoid, flavonoid, and anthocyanin biosynthesis pathways, respectively (Supplementary Table 3). Among them, 12, 1, 23, 39, and 2 unigenes were significantly upregulated in SG, SP, SY, SZ, and ST, respectively. For terpenoid biosynthesis-related genes, 73, 20, 14, 40, and 26 unigenes were involved in the terpenoid backbone, monoterpenoid, sesquiterpenoid and triterpenoid, diterpenoid, and tetraterpenoid biosynthesis pathways, respectively (Supplementary Table 4). Among them, 3, 1, 19, and 19 unigenes were significantly upregulated in SG, SP, SY, and SZ, respectively. A total of 133 transcription factors (TFs) reported to participate in the regulation of flavonoid and/or terpenoid biosynthesis were identified. These TFs included five *AP2*, seven *ERF*, 30 *MYB*, 62 *R2R3-MYB*, seven *bHLH*, 15 *WRKY*, six *ZIP*, and one *WD40* (Supplementary Table 5). Among them, 4, 2, 13, 2, and 2 were significantly upregulated in SG, SP, SY, SZ, and ST, respectively.

### Quantitative RT-PCR Validation of Differential Gene Expression

**Figure 4:**
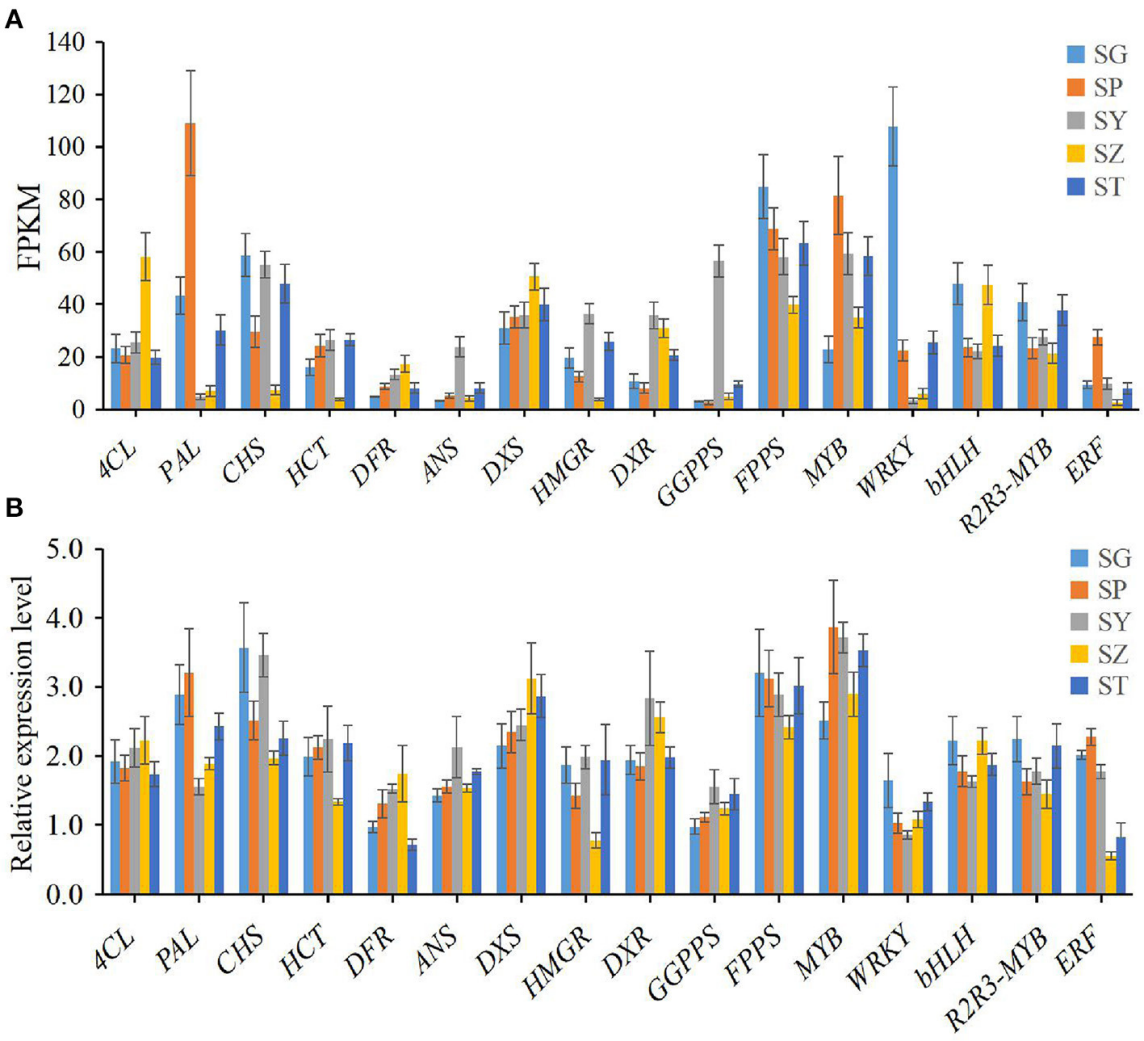
Expression patterns of 16 selected unigenes in different tissues of *Pinus taeda*. **(A)** The expression level of 16 unigenes is based on the fragments per kilobase of transcripts per million mapped fragments (FPKM) value. **(B)** The relative expression level of 16 unigenes obtained by quantitative real-time PCR (qRT-PCR) analysis. The error bars represent the SD from three replicates.

To verify the accuracy of the transcriptome data, the expression level of 16 candidate unigenes related to terpenoid and flavonoid biosynthesis was checked using qRT-PCR with three biological replicates tested. The primers of the 16 selected unigenes are listed in Supplementary Table 6. The expression levels of all 16 candidate unigenes differed in the SG, SP, SY, SZ, and ST tissues of *P. taeda*, and showed expression patterns similar to those of the transcriptome data (Figures 4A,B). Therefore, our RNA-seq and qRT-PCR analysis results showed high reliability and can be used for advanced research on related genes involved in terpenoid and flavonoid accumulation in different tissues of *P. taeda*.

### Metabolomic Profiling

**Figure 5:**
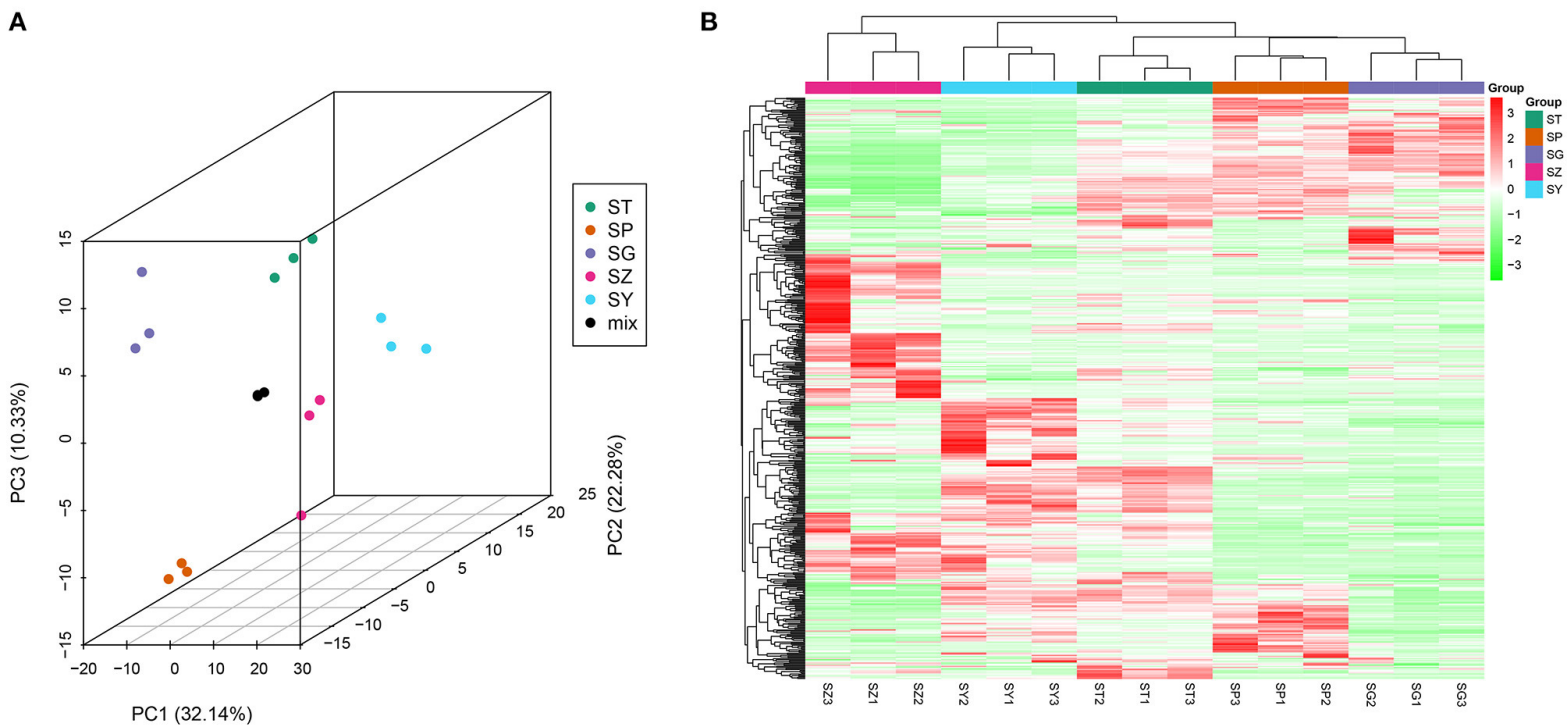
Differential metabolites analysis. **(A)** PCA 3D plot; **(B)** Heatmap based on hierarchical clustering analysis.

The total ion current (TIC) of all the samples showed high stability, large peak capacity, and good retention time (Supplementary Figure 4). A widely targeted metabolomic analysis was performed to produce a metabolic profile. A total of 528 metabolites were identified in the five tissues of *P. taeda* and were divided into 16 categories, primarily flavonoids, phenolic acids, and lipids, though only four terpenoids were detected (Table 2, Supplementary Table 7). Secondary metabolites comprised a large proportion of the detected metabolites, indicating that *P. taeda* possesses vigorous secondary metabolic activities. In this study, PCA was used to reveal the overall metabolite differences among the groups and the variability between the intragroup samples. PC1 and PC2 explained 32.14% and 22.28% of the total variance of the samples, respectively, and the accumulated contribution rate reached 54.42%. The results showed that the PCA clearly grouped all samples into distinct clusters, which suggested obvious metabolite differences among the different tissues of *P. taeda* (Figure 5A). The heatmap hierarchical clustering results also showed that the biological replicates grouped together, and the metabolite contents in SG, SP, SY, SZ, and ST varied greatly (Figure 5B). These results indicated that the correlation between replicates and the stability of the instrument was good and the metabolome data were highly reliable.

**Table 2:** Categories of the 528 metabolites identified in five tissues of *P. taeda*.

**Category**	**Number of metabolites**	**Category**	**Number of metabolites**
Flavonoids	168	Lignans	20
Phenolic acids	67	Proanthocyanidins	15
Lipids	55	Vitamin	10
Amino acids and derivatives	40	Coumarins	9
Nucleotides and derivatives	38	Stilbene	8
Organic acids	30	Others	7
Saccharides and alcohols	28	Tannin	5
Alkaloids	24	Terpenoids	4

### Identification of DAMs

**Figure 6:**
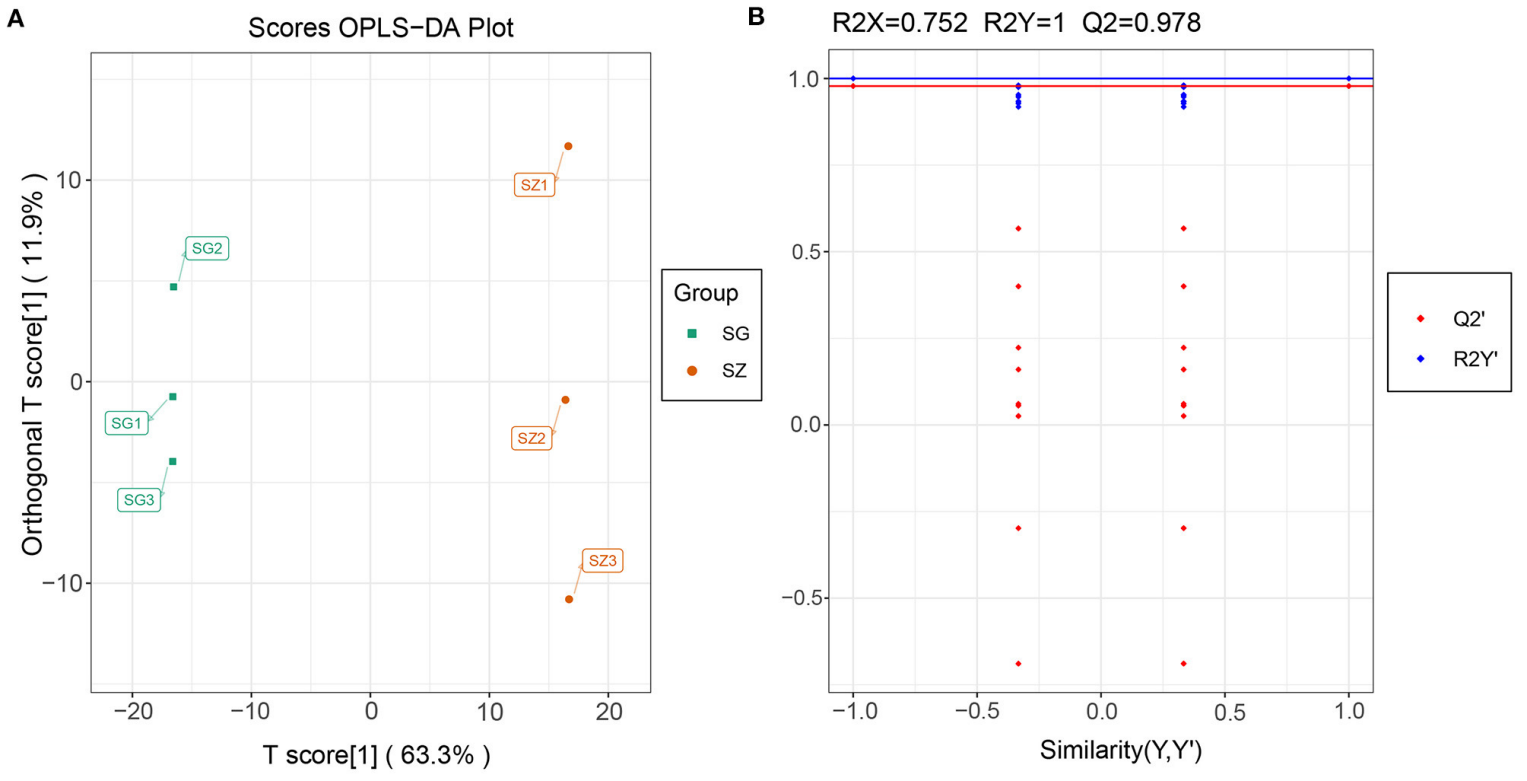
Metabolomics profiling of SG_vs_SZ group. **(A)** Orthogonal partial least squares-discriminant analysis (OPLS-DA); **(B)** The 200-response sorting tests of the OPLS-DA model, Q2 is an important parameter for evaluating the OPLS-DA model, and the R2X and R2Y represent the percentage of OPLS-DA model that can explain X and Y matrix information, respectively.

Differentially accumulated metabolites were identified for each comparison group by univariate and multivariate statistical analysis with threshold values of VIP ≥ 1 and fold change ≥ 2 or ≤ 0.5. The results of the OPLS-DA and the 200-response sorting tests are shown in Figure 6 and Supplementary Figure 5. The results indicated that the model was stable and reliable, and the VIP analysis could be used to screen the differential metabolites. A total of 493 DAMs were obtained from the comparison groups (Supplementary Table 8), and the DAMs in each comparison group are presented in Table 1 and Supplementary Table 9. The 3,7-Di-O-methyl quercetin (mws0917) was differentially accumulated in all of the comparison groups.

### Enrichment Analysis of the DAMs

**Figure 7:**
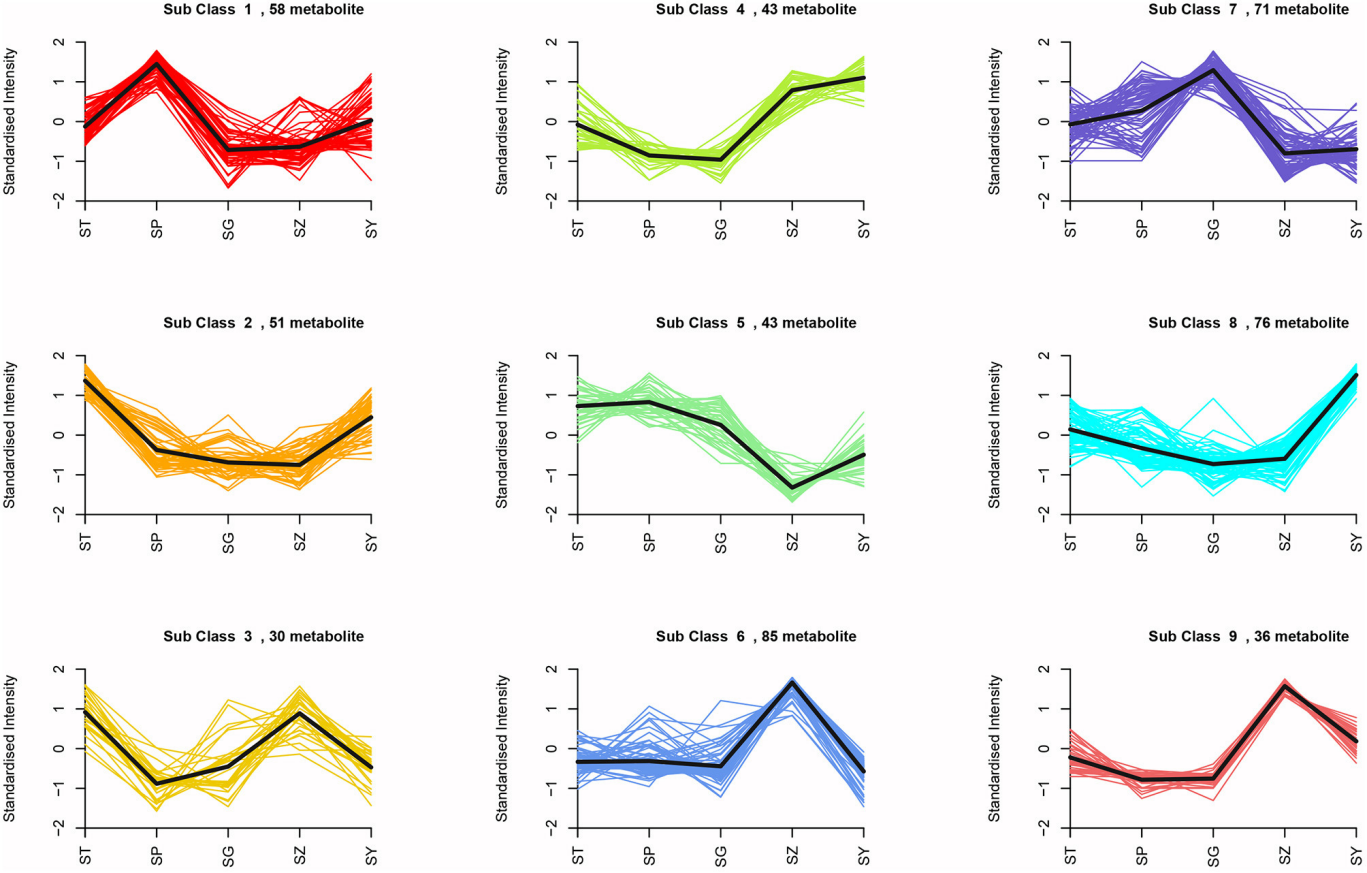
K-means analysis of differential metabolites.

means analysis divided the 493 DAMs into nine clusters (Figure 7). Among them, 58 metabolites were significantly increased in SP, and mainly included L-valine, L-leucine, L-isoleucine, dihydroquercetin, dihydromyricetin, quercetin, gallic acid, and free fatty acids. A total of 121 metabolites were significantly accumulated in SZ, including 53 flavonoids and 14 lignans. Seventy-one metabolites were significantly increased in SG, primarily, saccharides, alcohols, and proanthocyanidins (Supplementary Table 10). KEGG enrichment analysis was also performed to explore the functional classification of the DAMs obtained from each comparison group. The results showed that the DAMs in each comparison group were enriched in similar pathways, such as the phenylpropanoid, flavonoid, flavone and flavonol, and amino acid biosynthesis pathways.

### Correlation Analysis Between Transcriptome and Metabolome Data

**Figure 8:**
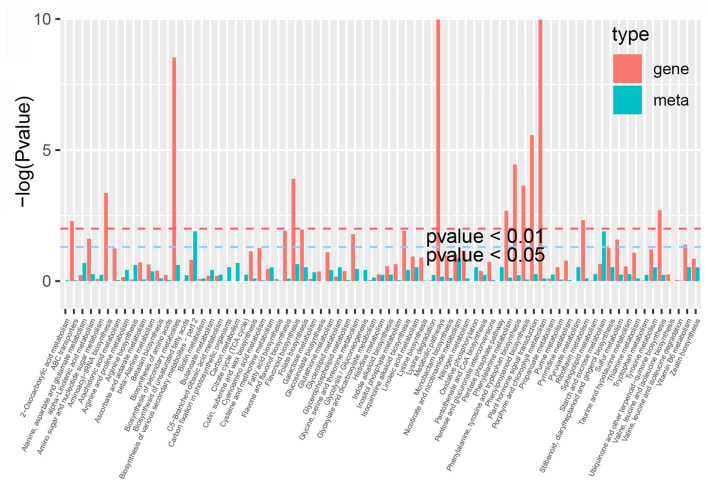
KEGG enrichment analysis of the differentially expressed genes (red column) and differentially accumulated metabolites (green column) that were enriched in the same pathway.

By comparing the DEGs and DAMs in each group, we observed that many DEGs and DAMs were enriched in the same KEGG pathway. These pathways mainly included the phenylpropanoid, flavonoid, flavone and flavonol, and aminoacyl-tRNA, and amino acids biosynthesis pathways, as well as the metabolic pathways of 2-oxocarboxylic acid, pyrimidine, glycine, serine and threonine, and ABC transporters (Figure 8, Supplementary Table 11). For example, a total of 59 DEGS and 28 DAMs were enriched in the same flavonoid biosynthesis pathway in *P. taeda*.

**Figure 9:**
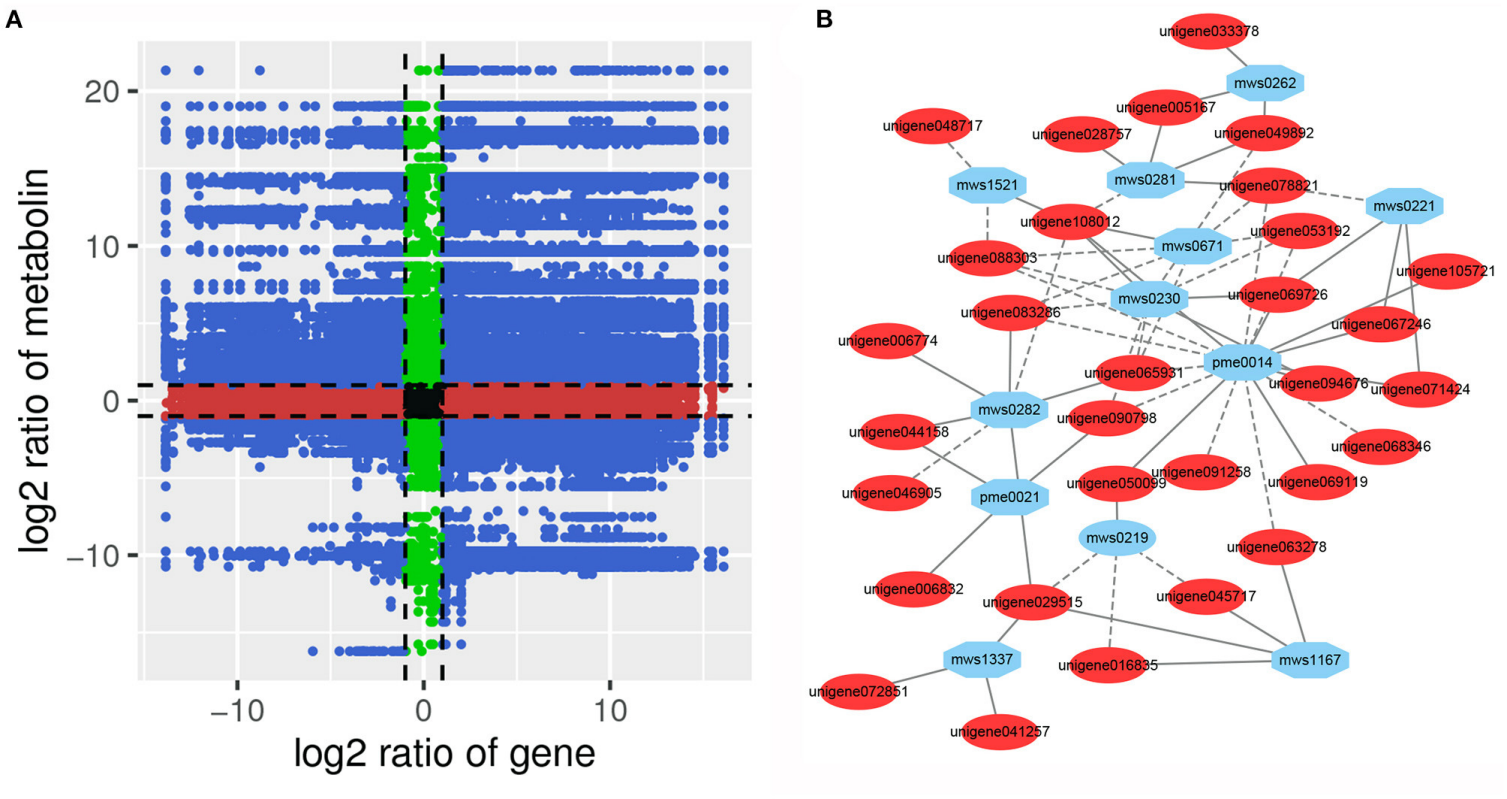
**(A)** Nine quadrant diagram showing the correlation of differentially expressed genes and differentially accumulated metabolites between SG and SZ libraries. **(B)** Connection network between differentially expressed genes (blue ovals) and differentially accumulated metabolites (red ovals).

To analyze the regulatory network of the DAMs, correlation analysis of the DEGs and DAMs was performed in each group. Many metabolites were positively or negatively regulated by multiple genes (Figure 9A, Supplementary Figure 6). Correlation analysis showed that a total of 219 DEGs were significantly correlated with 45 DAMs (Supplementary Table 12). Among them, a single gene was regulated by multiple metabolites, or a single metabolite was regulated by multiple genes that are ubiquitous. For example, L-threonine (mws0230) and L-glutamic acid (pme0014) were significantly correlated with 44 DEGs (29 negatively correlated, 15 positively correlated) and 129 DEGs (106 negatively correlated, 23 positively correlated), respectively. The DEG denoted as unigene108012 and annotated as pyruvate kinase [EC:2.7.1.40] was positively correlated with L-threonine, L-glutamic acid, L-homoserine (mws0671), and salicin (mws1521), and negatively correlated with L-tryptophan (mws0282), citric acid (mws0281), and ademine (pme0040), respectively. However, the unigene088303 annotated as phosphoglycerate kinase [EC:2.7.2.3] showed a strong negative correlation (PCC > 0.90) with mws0671, pme0014, mws0230, and mws1521 (Figure 9B). In addition, five DEGs were significantly correlated with six flavonoids. Among them, two cytochrome P450 (CYP) family genes CYP75B91 and CYP75B95 were significantly positively correlated with dihydrochrysin, pinostrobin, kaempferol 3-O-β-D-sophoroside, and nicotiflorin. These results indicate that a complex regulatory mechanism exists between the variation in metabolite accumulation and gene expression abundance in *P. taeda*.

## Discussion

### Transcriptome Analysis of *P. taeda*

Transcriptome sequencing is now a routine experimental method for novel gene discovery, quantitative gene expression, and transcript identification. For instance, numerous genes related to volatile terpenoids have been identified by transcriptome sequencing in *Citrus medica* var. s*arcodactylis* (Xu et al., 2019) and *Picea abies* (Yassaa et al., 2012). Although some terpenoid biosynthesis-related genes were previously identified in the secondary xylem of *P. taeda* (Mao et al., 2019), the flavonoid and terpenoid biosynthesis-related genes in different tissues of *P. taeda* had not been elucidated. In this study, transcriptome analysis was performed on five tissues of *P. taeda*. A total of 108,663 unigenes were assembled, of which 39,576 unigenes obtained functional annotations from the UniProt, Nr, GO, KOG, and KEGG databases, which is more than those obtained in the RNA-Seq results for the secondary xylem of *P. taeda* (Mao et al., 2019). These results indicated that numerous unigenes have tissue-specific expression. A total of 14,282 unigenes exhibited significant homology with sequences from *Picea sitchensis*. However, very few unigenes matched the sequences from *P. taeda*. One possible explanation for this is the lack of available genomic information for *P. taeda* in public databases. The differential expression analysis showed that the number of DEGs in the SZ vs. SP, SZ vs. SY, SZ vs. SG, and SZ vs. ST groups all exceeded 7,000. In addition, pathway analysis revealed that the DEGs were mainly enriched in the metabolic pathway and biosynthesis of the secondary metabolites pathway. These results suggest that more extensive metabolic activity may have occurred in the SZ of *P. taeda*.

A total of 343 and 173 candidate genes related to flavonoid and terpenoid biosynthesis were identified, respectively. Among them, 77 (22.45%) flavonoid biosynthesis-related genes and 42 (24.28%) terpenoid biosynthesis-related genes were significantly upregulated in the various tissues of *P. taeda*. SZ possessed the largest number of significantly upregulated genes. These results indicated that the SZ tissue of *P. taeda* is associated with higher flavonoid and terpenoid metabolic activity, which is consistent with the results of the KEGG pathway analysis of the DEGs. The genes in the flavonoid and terpenoid pathways are subjected to regulation at the transcriptional level (Winkel-Shirley, 2001). Previous studies have revealed that several TFs, such as *R2R3-MYB, bHLH*, and *WD40* participate in flavonoid biosynthesis pathways (Allan et al., 2008; Palapol et al., 2009), and *AP2, ERF, bHLH, WRKY*, and *ZIP* are all key genes contributing to the terpenoid secondary metabolic pathway (Dai et al., 2009; Cheng et al., 2012). In this study, a total of 133 TFs that have been reported to participate in the regulation of flavonoid and/or terpenoid biosynthesis pathways were identified. These results help elucidate the regulatory factors involved in flavonoid and terpenoid biosynthesis in *P. taeda*.

### Metabolome Analysis of *P. taeda*

In addition to being a major afforestation tree, *P. taeda* is also a potential biofuel and source for products. The main active materials of pine extracts are terpenoids and flavonoids. Many monoterpenoids, sesquiterpenoids, and diterpenoids have been identified in *P. taeda* and other *Pinus* species by gas chromatography-mass spectrometry (GC-MS) analysis (Tsitsimpikou et al., 2001). However, less attention has been given to the flavonoids and other active substances in the extracts. Plant metabolomics, as a relatively new field in the postgenome era, has been effectively applied to evaluate the changes in metabolites among various tissues, species, or developmental phases (Kaiser, 2012; Li et al., 2018). In this study, the metabolites in five tissues of *P. taeda* were systematically analyzed and identified using a widely targeted UPLC–MS/MS metabolomics approach. A total of 528 metabolites were identified, 168 of which were flavonoids. This result showed that *P. taeda* exhibits strong flavonoid metabolic activity, which is consistent with the results of the transcriptome analysis. However, only four non-volatile terpenoids were detected. This suggests that UPLC-MS/MS cannot be easily used to systematically identify the structure of non-volatile polyterpenes in the absence of standards. In addition, a total of 121 metabolites significantly accumulated in the SZ of *P. taeda*, thereby providing a theoretical basis for product development using pine needles as a raw material.

### Integrated Analysis of the Transcriptome and Metabolome

Metabolites are the intermediate or final products of the cell biological regulation process, and their accumulation level strongly regulates plant growth and development (Pichersky and Lewinsohn, 2011; De Luca et al., 2012). Their accumulation is controlled by many exogenous and endogenous factors. Therefore, metabolomics analysis is typically used as a technical means of association analysis together with transcriptomics to identify functional genes and elucidate the metabolic pathway of interest (Matus, 2016; Wang et al., 2017; Dong et al., 2019). We used an integrated analysis of the transcriptome and metabolome to show that many DEGs and DAMs were involved in the same flavonoid and amino acid biosynthesis pathways. This suggests that *P. taeda* exhibits strong flavonoid and amino acid metabolic activity. A total of 219 DEGs were significantly correlated with 45 DAMs in *P. taeda*. For example, the accumulation level of L-glutamic acid (pme0014) was positively correlated with 23 DEGs and negatively correlated with 106 DEGs. The expression level of unigene108012 was significantly correlated with the accumulation level of seven metabolites. These results indicate that there is a complex regulatory mechanism between the variation in metabolite accumulation and gene expression abundance in *P. taeda*. However, few DEGs that were significantly correlated with flavonoid accumulation have been identified. To improve understanding of the molecular mechanism of flavonoid accumulation in *P. taeda*, further studies are required to elucidate the correlation between different numbers of flavonoid biosynthetic genes in different stages with their tissue accumulation. Interestingly, two CYP family genes, namely CYP75B91 and CYP75B95 were found to be significantly positively correlated with four flavonoids in *P. taeda* for the first time. This suggests that the CYP gene family might play an important role in flavonoid accumulation in *P. taeda*. Overall, these results illustrate the correlation between metabolites and genes in *P. taeda* and provide a basis for understanding the genetic regulation mechanism of the metabolite changes in *P. taeda*.

## Data Availability Statement

Our RNA-seq data of 15 cDNA libraries have been deposited in the repositories of NCBI Sequence Read Archine with Bioproject accession number: PRJNA698255 and SRA accession number: SRR1367098–SRR1367112.

## Author Contributions

TL conceived the study, participated in its design and coordination, performed the experimental measurements, processed the experimental data, interpreted the data, and drafted and revised the manuscript. JM participated in study design and coordination, performed the experimental measurements, processed the experimental data, interpreted the data, and drafted and revised the manuscript. LH and MC performed the experimental measurements and helped in sampling. WZ and ZF processed the experimental data and helped in sampling. SH participated in the design and helped in drafting the manuscript. All authors contributed to the article and approved the submitted version.

## Conflict of Interest

The authors declare that the research was conducted in the absence of any commercial or financial relationships that could be construed as a potential conflict of interest.

## Publisher's Note

All claims expressed in this article are solely those of the authors and do not necessarily represent those of their affiliated organizations, or those of the publisher, the editors and the reviewers. Any product that may be evaluated in this article, or claim that may be made by its manufacturer, is not guaranteed or endorsed by the publisher.
