# Supplementary material for: Integrated Analysis of the Transcriptome and Metabolome Reveals Genes Involved in Terpenoid and Flavonoid Biosynthesis in the Loblolly Pine (Pinus taeda L.)
Source: Front Plant Sci. 2021 Oct 1;12:729161. doi: 10.3389/fpls.2021.729161 (PMC8519504; doi:10.3389/fpls.2021.729161)
Supplement: Supplementary file 1 [file Data_Sheet_1.ZIP › Supplementary Figure 4.pdf]

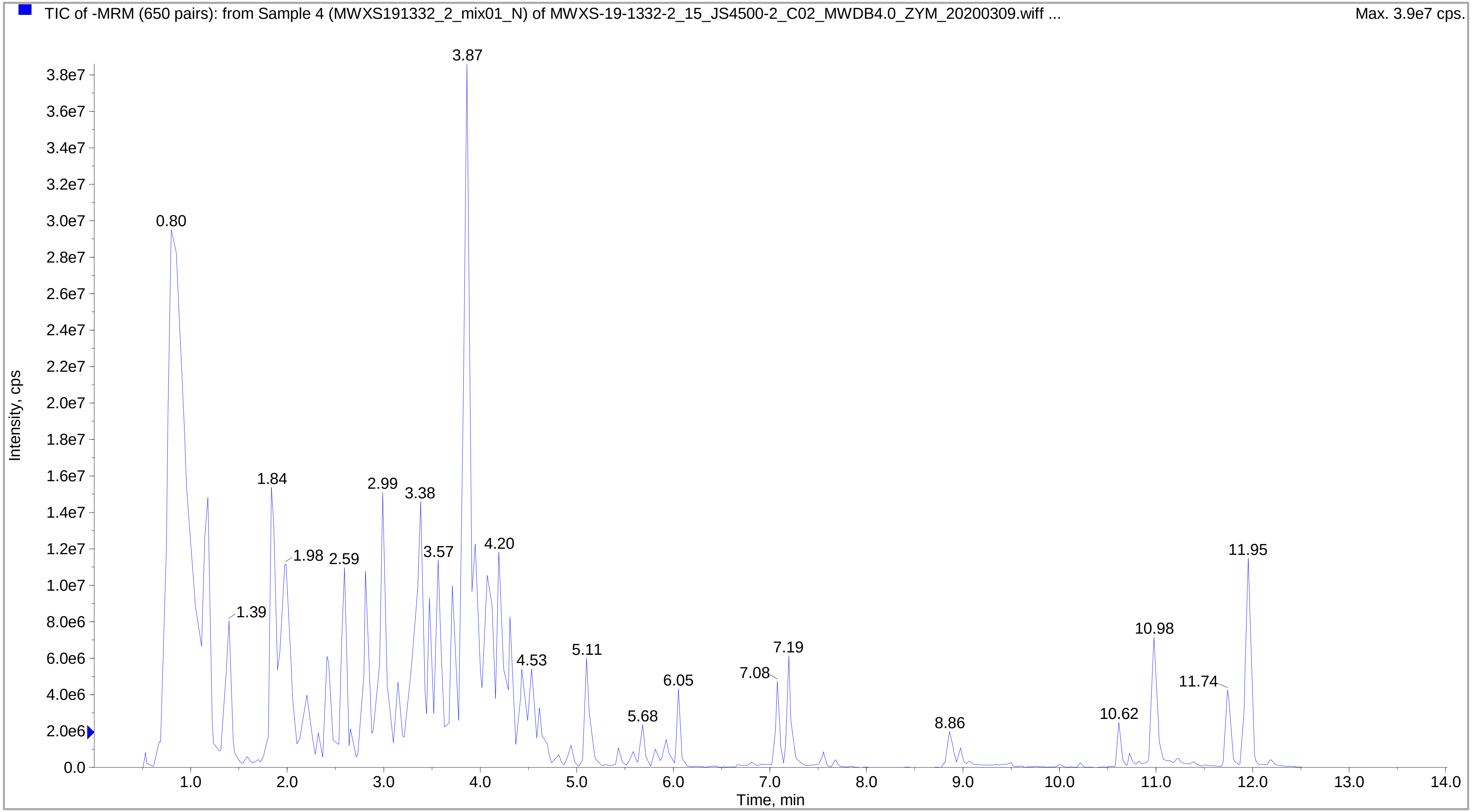

Supplementary Figure 4-1 Total Ion Current of all samples in negative ion mode

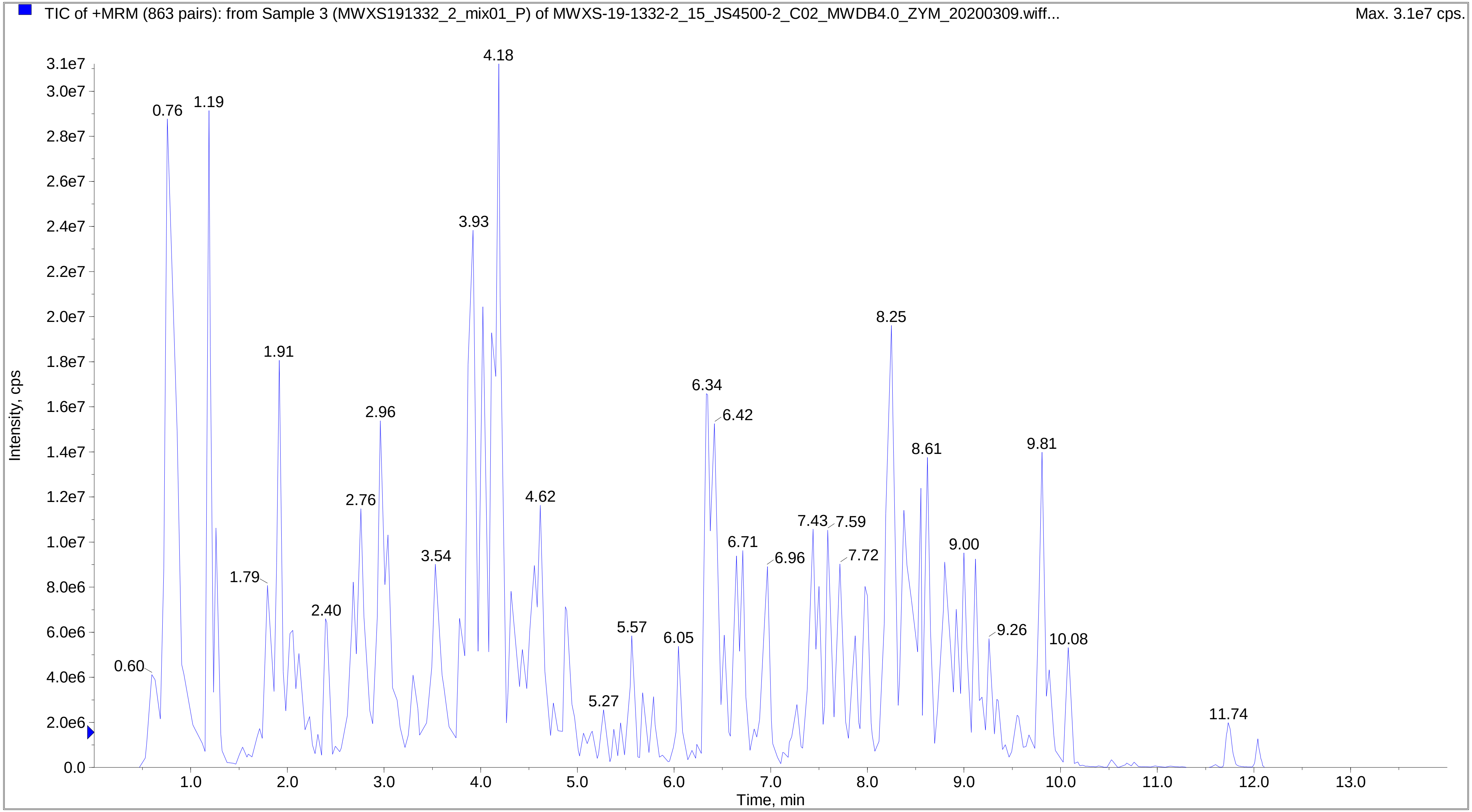

Supplementary Figure 4-2 Total Ion Current of all samples in positive ion mode
