# Supplementary material for: Integrated Analysis of the Transcriptome and Metabolome Reveals Genes Involved in Terpenoid and Flavonoid Biosynthesis in the Loblolly Pine (Pinus taeda L.)
Source: Front Plant Sci. 2021 Oct 1;12:729161. doi: 10.3389/fpls.2021.729161 (PMC8519504; doi:10.3389/fpls.2021.729161)
Supplement: Supplementary file 1 [file Data_Sheet_1.ZIP › Supplementary Figure 5.pdf]

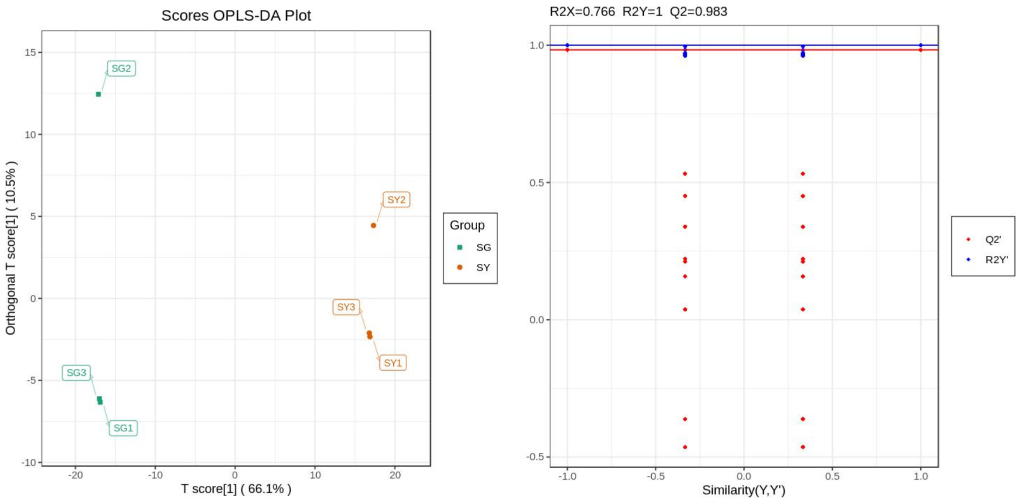

Supplementary Figure 5-1. OPLS-DA and the 200-response sorting tests of SG_vs_SY group

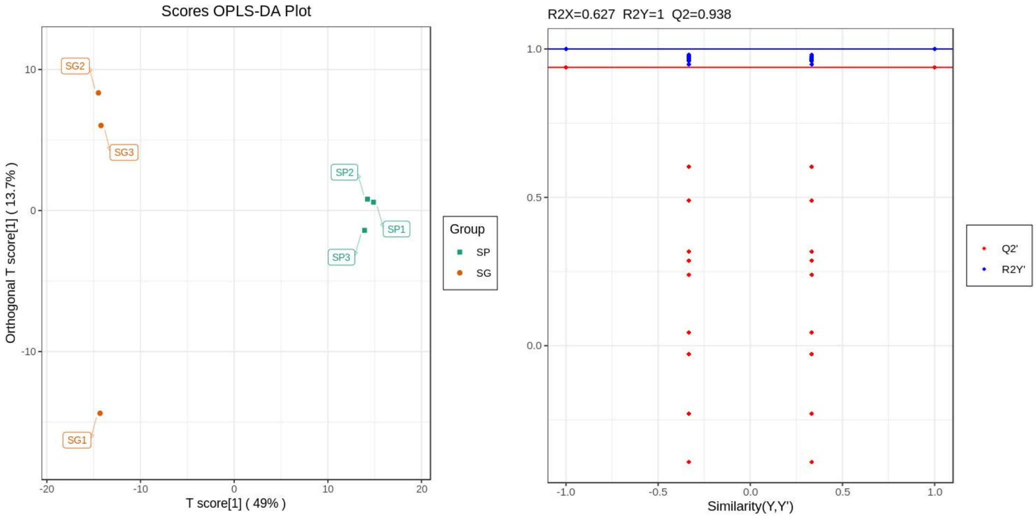

Supplementary Figure 5-2. OPLS-DA and the 200-response sorting tests of SG_vs_SP group

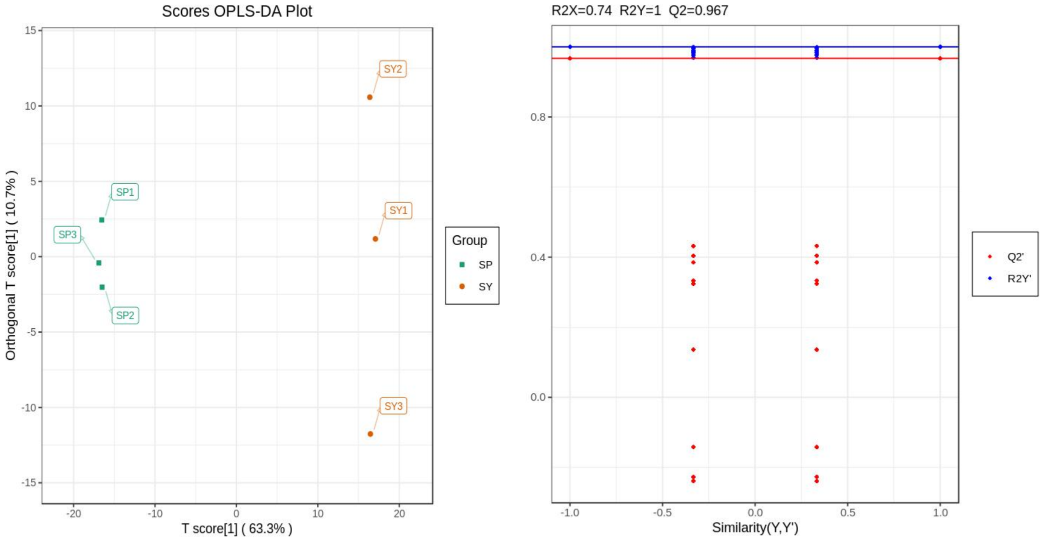

Supplementary Figure 5-3. OPLS-DA and the 200-response sorting tests of SY_vs_SP group

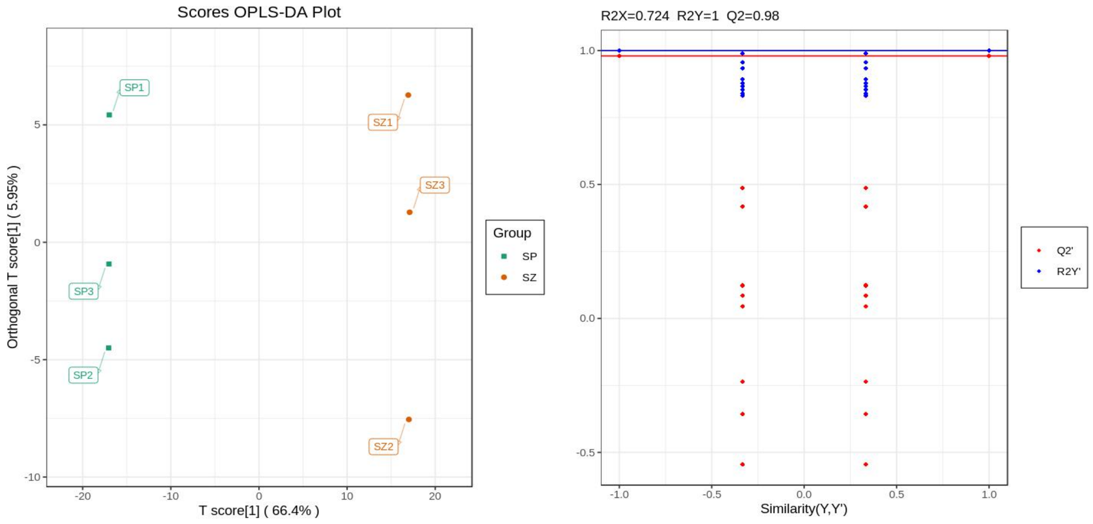

Supplementary Figure 5-4. OPLS-DA and the 200-response sorting tests of SZ_vs_SP group

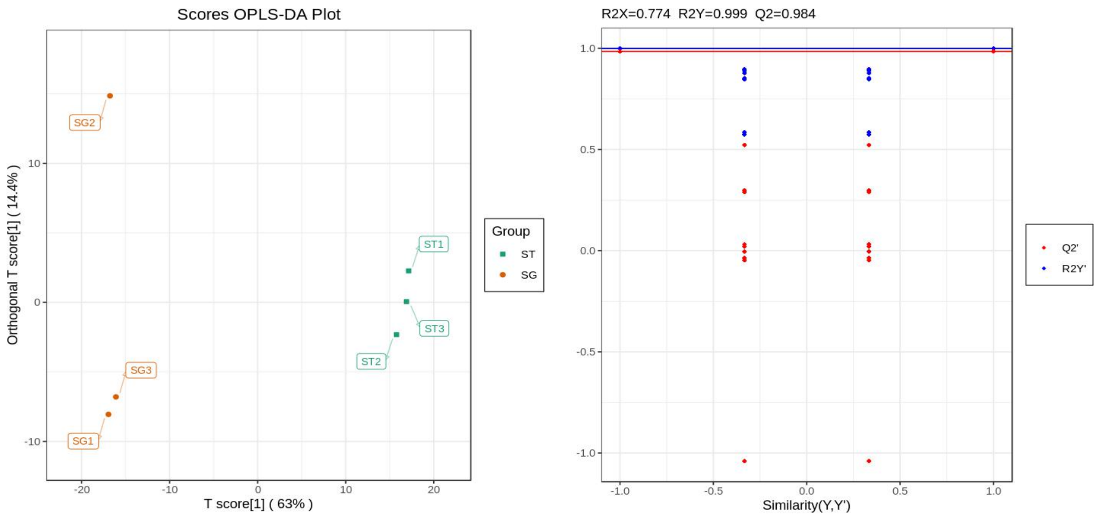

Supplementary Figure 5-5. OPLS-DA and the 200-response sorting tests of ST_vs_SG group

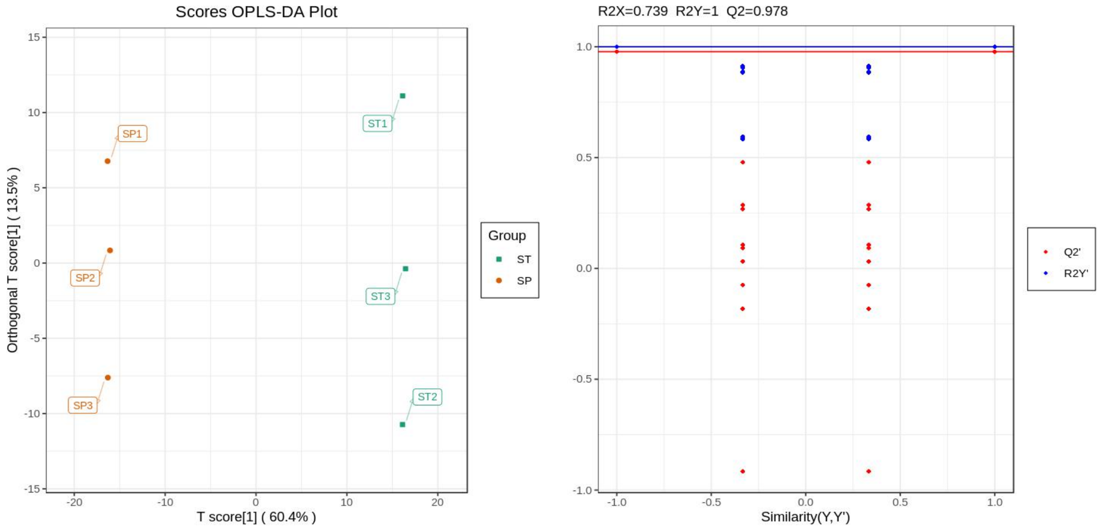

Supplementary Figure 5-6. OPLS-DA and the 200-response sorting tests of ST_vs_SP group

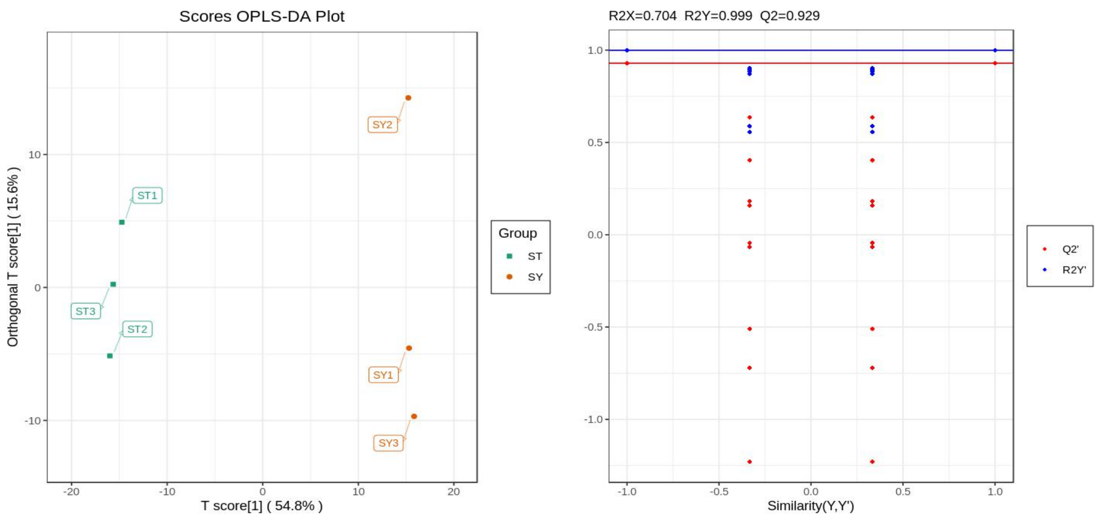

Supplementary Figure 5-7. OPLS-DA and the 200-response sorting tests of SY_vs_ST group

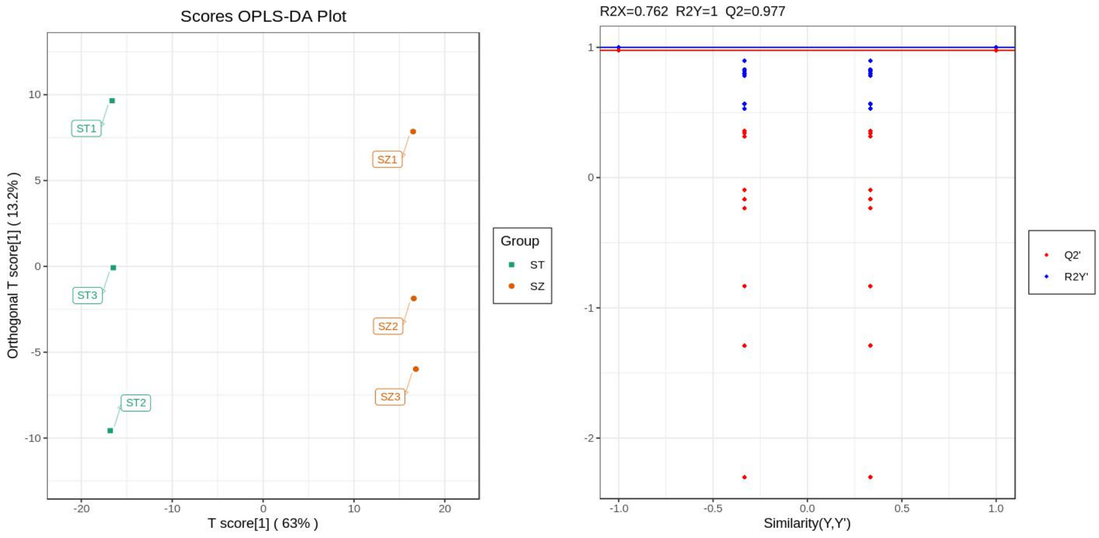

Supplementary Figure 5-8. OPLS-DA and the 200-response sorting tests of ST_vs_SZ group

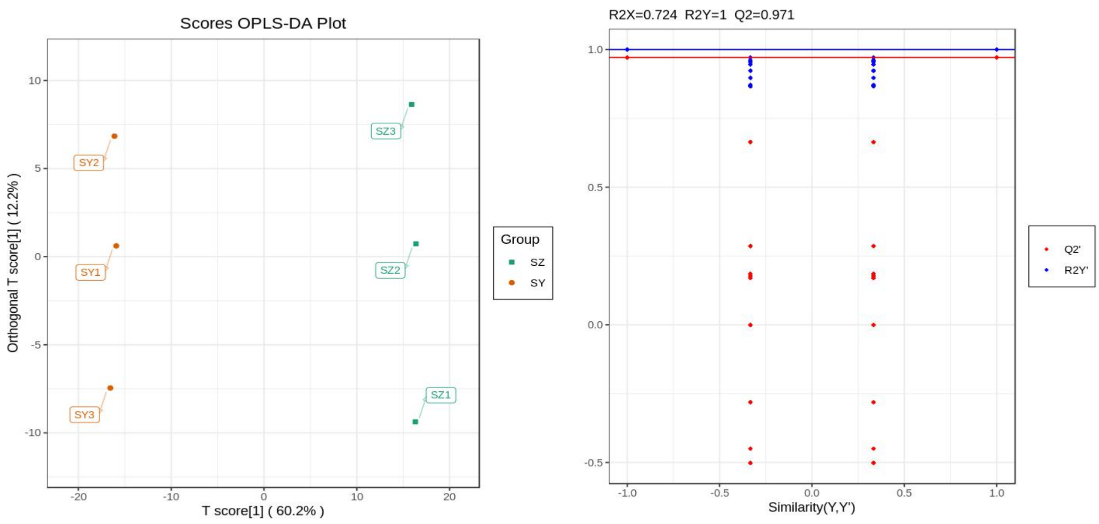

Supplementary Figure 5-9. OPLS-DA and the 200-response sorting tests of SY_vs_SZ group
