# Supplementary material for: Integrated Analysis of the Transcriptome and Metabolome Reveals Genes Involved in Terpenoid and Flavonoid Biosynthesis in the Loblolly Pine (Pinus taeda L.)
Source: Front Plant Sci. 2021 Oct 1;12:729161. doi: 10.3389/fpls.2021.729161 (PMC8519504; doi:10.3389/fpls.2021.729161)
Supplement: Supplementary file 1 [file Data_Sheet_1.ZIP › Supplementary Figure 6.pdf]

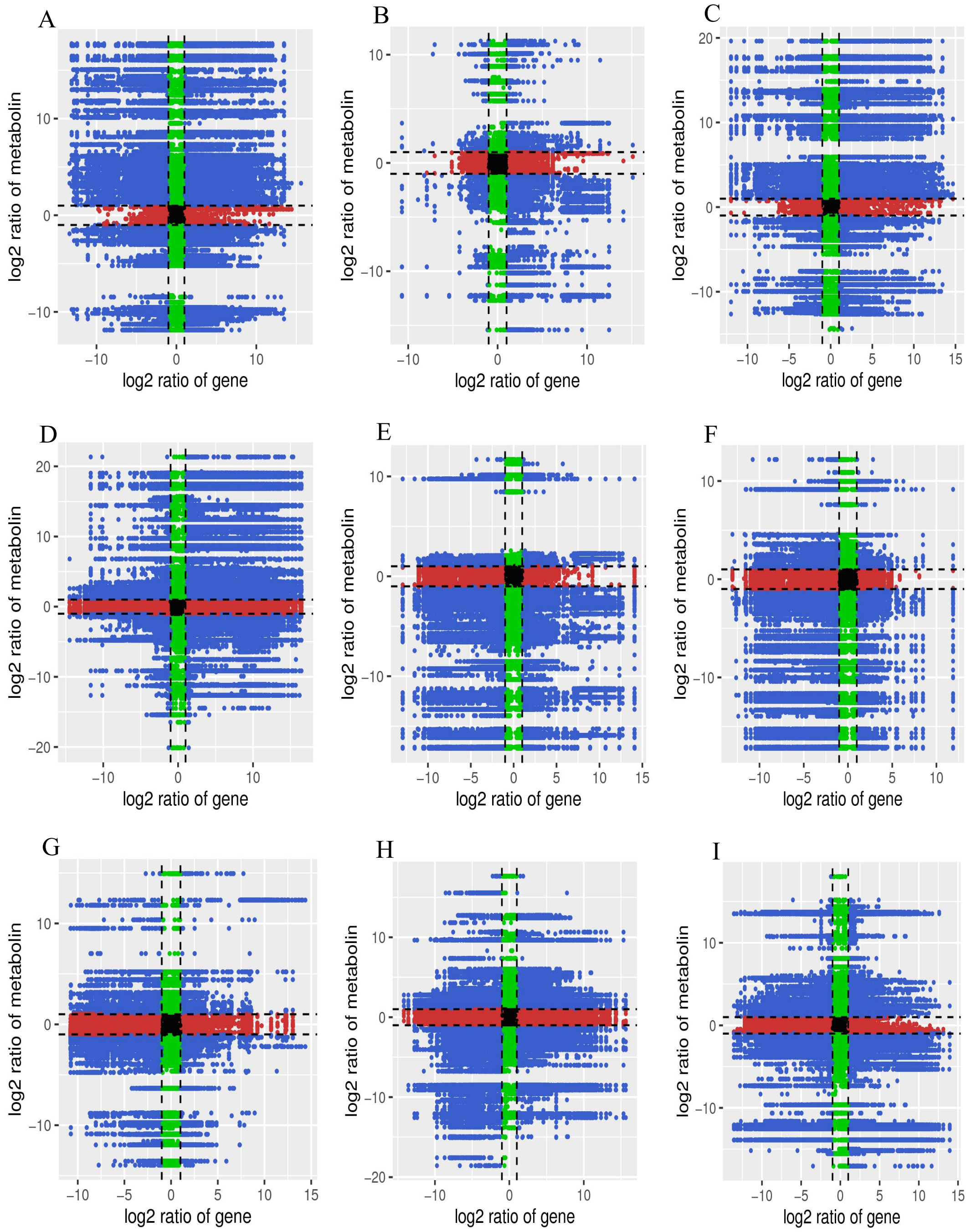

Supplementary Figure 6. The nine quadrant diagram showing the correlation of differentially expressed genes and differentially accumulated metabolites from each groups of *Pinus taeda*. A, B, C, D, E, F, G, H and I represented the groups of SG_vs_SY, SP_vs_SG, SP_vs_SY, SP_vs_SZ, ST_vs_SG, ST_vs_SP, ST_vs_SY, ST_vs_SZ and SZ_vs_SY, respectively. The blue, red, green and black points indicate the differentially expressed gene-differentially expressed metabolite pairs, differentially expressed gene-non-differentially expressed metabolite pairs, non-differentially expressed gene-differentially expressed metabolite pairs, and non-differentially expressed gene-non-differentially expressed metabolite pairs, respectively.
