# Supplementary figures and images for: Integrated Analysis of the Transcriptome and Metabolome Reveals Genes Involved in Terpenoid and Flavonoid Biosynthesis in the Loblolly Pine (Pinus taeda L.)
Source: Front Plant Sci. 2021 Oct 1;12:729161. doi: 10.3389/fpls.2021.729161 (PMC8519504; doi:10.3389/fpls.2021.729161)

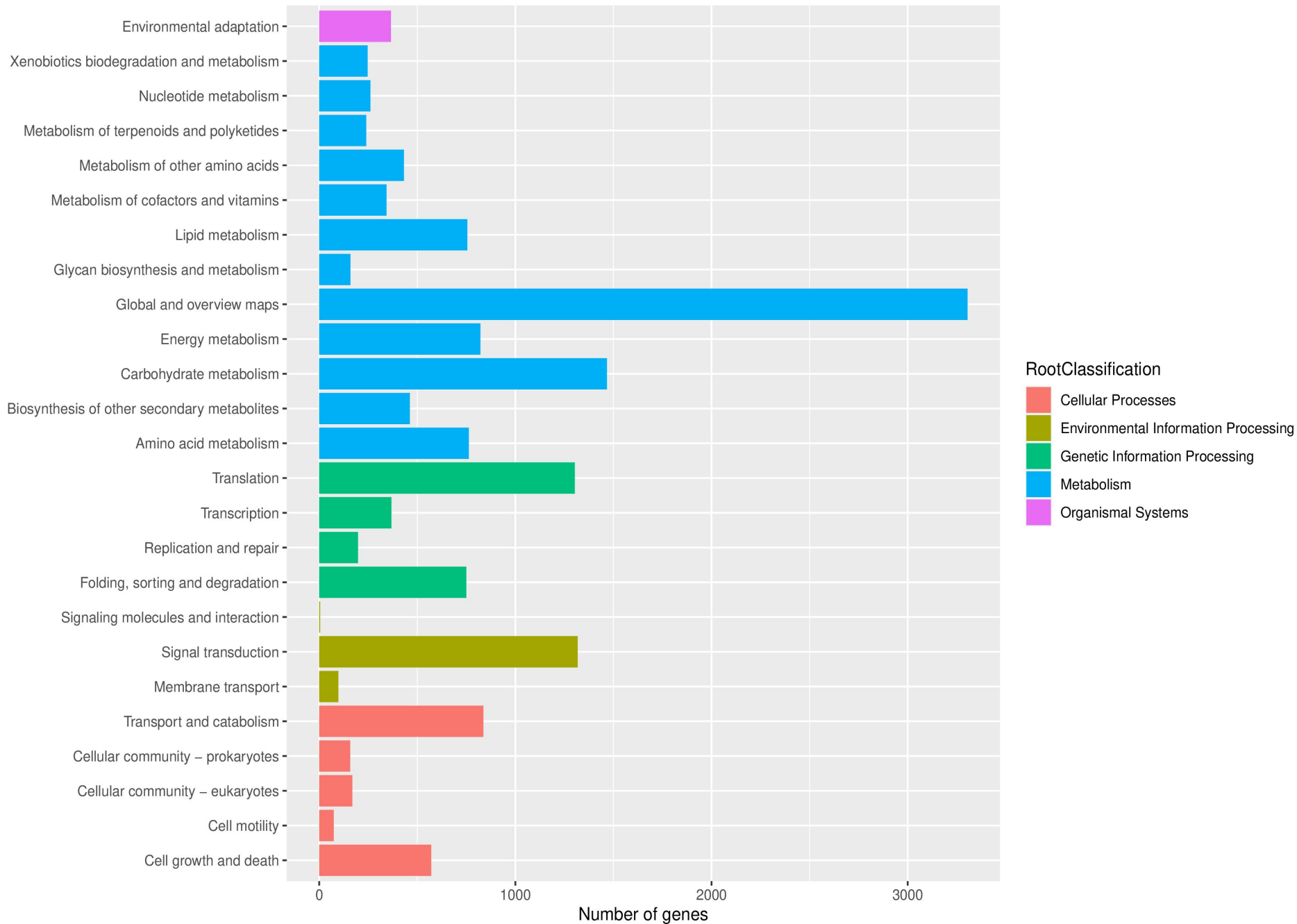

Supplementary Figure 2 The KEGG enrichment analysis of 39,576 annotated unigenes

Supplement: Supplementary file 1 [file Data_Sheet_1.ZIP › Supplementary Figure 2.pdf]

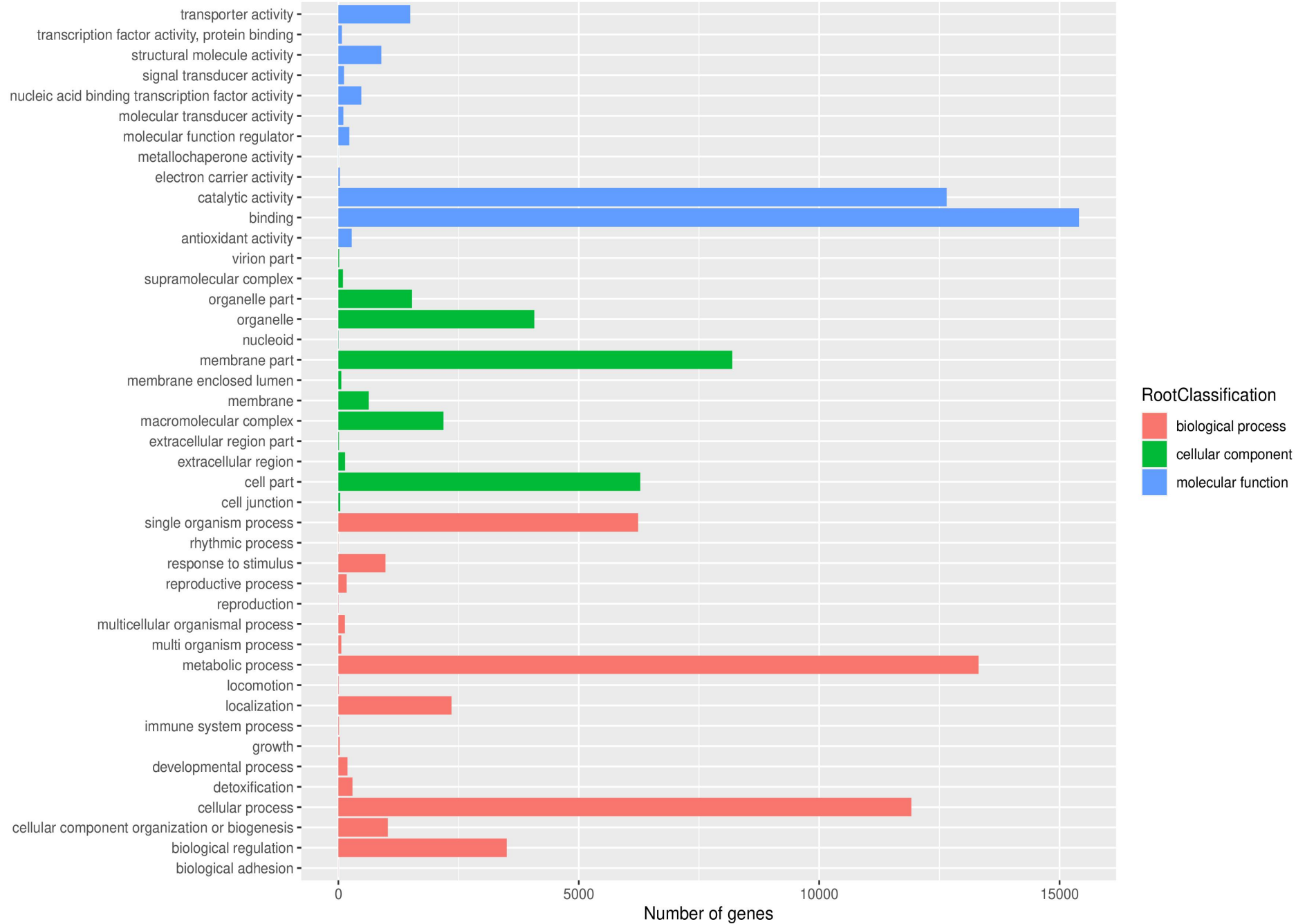

Supplementary Figure 1 The GO enrichment analysis of 39,576 annotated unigenes

Supplement: Supplementary file 1 [file Data_Sheet_1.ZIP › Supplementary Figure 1.pdf]
