# Supplementary material for: Integrated Analysis of the Transcriptome and Metabolome Reveals Genes Involved in Terpenoid and Flavonoid Biosynthesis in the Loblolly Pine (Pinus taeda L.)
Source: Front Plant Sci. 2021 Oct 1;12:729161. doi: 10.3389/fpls.2021.729161 (PMC8519504; doi:10.3389/fpls.2021.729161)
Supplement: Supplementary file 2 [file Data_Sheet_2.ZIP › Supplementary Table 1.doc]

*Table S1 Summary of the sequencing quality of 15 cDNA libraries of Pinus taeda*

| **Sample** | **ReadsNum** | **BaseNum** | **TotalBase** | **GC** | **Q20** | **Q30** |
| --- | --- | --- | --- | --- | --- | --- |
| SG_1 | 34,366,844 | 5,155,026,600 | 10,310,053,200 | 45.26% | 98.27% | 95.08% |
|  | 34,366,844 | 5,155,026,600 |  | 45.13% | 97.09% | 92.83% |
| SG_2 | 24,222,866 | 3,633,429,900 | 7,266,859,800 | 45.19% | 98.07% | 94.47% |
|  | 24,222,866 | 3,633,429,900 |  | 44.95% | 96.62% | 91.73% |
| SG_3 | 24,144,398 | 3,621,659,700 | 7,243,319,400 | 44.72% | 98.18% | 94.74% |
|  | 24,144,398 | 3,621,659,700 |  | 44.38% | 96.54% | 91.50% |
| SP_1 | 26,529,511 | 3,979,426,650 | 7,958,853,300 | 45.30% | 98.07% | 94.37% |
|  | 26,529,511 | 3,979,426,650 |  | 45.25% | 96.10% | 90.47% |
| SP_2 | 28,938,707 | 4,340,806,050 | 8,681,612,100 | 46.96% | 98.21% | 94.69% |
|  | 28,938,707 | 4,340,806,050 |  | 46.75% | 96.76% | 91.75% |
| SP_3 | 28,274,142 | 4,241,121,300 | 8,482,242,600 | 45.40% | 98.09% | 94.48% |
|  | 28,274,142 | 4,241,121,300 |  | 45.13% | 96.52% | 91.56% |
| SY_1 | 34,563,627 | 5,184,544,050 | 10,369,088,100 | 44.49% | 98.22% | 94.69% |
|  | 34,563,627 | 5,184,544,050 |  | 44.21% | 96.65% | 91.57% |
| SY_2 | 23,298,214 | 3,494,732,100 | 6,989,464,200 | 45.48% | 98.11% | 94.46% |
|  | 23,298,214 | 3,494,732,100 |  | 45.22% | 96.66% | 91.69% |
| SY_3 | 25,886,814 | 3,883,022,100 | 7,766,044,200 | 46.65% | 98.31% | 94.86% |
|  | 25,886,814 | 3,883,022,100 |  | 46.42% | 96.58% | 91.32% |
| SZ_1 | 24,511,341 | 3,676,701,150 | 7,353,402,300 | 45.55% | 98.36% | 94.99% |
|  | 24,511,341 | 3,676,701,150 |  | 45.23% | 96.85% | 91.96% |
| SZ_2 | 23,137,361 | 3,470,604,150 | 6,941,208,300 | 45.86% | 98.37% | 95.11% |
|  | 23,137,361 | 3,470,604,150 |  | 46.05% | 96.25% | 91.30% |
| SZ_3 | 28,554,873 | 4,283,230,950 | 8,566,461,900 | 46.74% | 98.32% | 94.97% |
|  | 28,554,873 | 4,283,230,950 |  | 46.37% | 96.66% | 91.72% |
| ST_1 | 24,778,248 | 3,716,737,200 | 7,433,474,400 | 46.83% | 98.37% | 95.01% |
|  | 24,778,248 | 3,716,737,200 |  | 46.54% | 97.03% | 92.37% |
| ST_2 | 26,378,350 | 3,956,752,500 | 7,913,505,000 | 45.14% | 98.11% | 94.40% |
|  | 26,378,350 | 3,956,752,500 |  | 45.00% | 96.10% | 90.47% |
| ST_3 | 26,429,748 | 3,964,462,200 | 7,928,924,400 | 45.32% | 98.19% | 94.68% |
|  | 26,429,748 | 3,964,462,200 |  | 45.05% | 96.83% | 91.93% |
