# Supplementary material for: Integrated Analysis of the Transcriptome and Metabolome Reveals Genes Involved in Terpenoid and Flavonoid Biosynthesis in the Loblolly Pine (Pinus taeda L.)
Source: Front Plant Sci. 2021 Oct 1;12:729161. doi: 10.3389/fpls.2021.729161 (PMC8519504; doi:10.3389/fpls.2021.729161)
Supplement: Supplementary file 2 [file Data_Sheet_2.ZIP › Supplementary Table 6.doc]

Supplementary Table 6 Primers of candidate genes for qRT-PCR analysis

| Gene | Forward and Reverse primer sequences | Length | Annealing (℃) |
| --- | --- | --- | --- |
| *WRKY* | F:CCACAATCACGCTCGACCTCAC | 22 | 56.2 |
|  | R:ACTGTTGGAATCTGCCTGCTGTC | 23 | 55.8 |
| *HMGR* | F:GTGAGGAGGAGGTCCTTGGAGATG | 24 | 54.0 |
|  | R:ACAGGCAGTTCACAGCATTGGC | 22 | 53.0 |
| *DXR* | F:CCTGCGACTCTGCCATCTTCAC | 22 | 55.0 |
|  | R:AGGCAGGAGGAGGTGCTTGTAG | 22 | 54.5 |
| *FPPS* | F:GTGCTTGGCTGGTGTATTGAATGG | 24 | 54.0 |
|  | R:AACAAGGTTGTCCACGCCTAGTG | 23 | 53.5 |
| *4CL* | F:TTGGATCGGCAGGAGCATTAGTTC | 24 | 59.4 |
|  | R:GCCACACTTCTCCTCGCTGATTAG | 24 | 60.1 |
| *ANS* | F:GATTCTGCGGACGAGGACATCAAG | 24 | 60.2 |
|  | R:TGGTTCAGCAACTGCATGACTCC | 23 | 60.2 |
| *CHS* | F:CGACAGGCGATGTTGGCTATGG | 22 | 61.4 |
|  | R:TCAAAGTCTGCTCCGGGTAGATCC | 24 | 60.8 |
| *DFR* | F:CTCGGGATTTCGCAGTGGAGAAC | 23 | 60.7 |
|  | R:GCCAAGGCAGTGATCATGCTAGG | 23 | 61.0 |
| *HCT* | F:GGCAGCCCCGATTTCTTTTCAATG | 24 | 59.5 |
|  | R:GGACACCCTCACCGTTACAGTTG | 23 | 60.5 |
| *PAL* | F:ATGACTCCGAGGACAGACCACAC | 23 | 61.2 |
|  | R:TGAATCCGTTGAGGCACTGAAGC | 23 | 60.2 |
| *ERF* | F:TAGAGGTATCAGGCAGCGTCCAC | 23 | 61.1 |
|  | R:CAAGCCAAACCATAGTGCCCTTTC | 24 | 59.0 |
| *BHLH* | F:ACTCAGACAATGCCCACGACAATG | 24 | 59.9 |
|  | R:TCGCTGCCATTCTGTCCAAACTC | 23 | 60.2 |
| *MYB* | F:GGGCTGCGAAGATGTGGAAAGAG | 23 | 61.1 |
|  | R:CGACCATCTGTTGCCCAAGAGAG | 23 | 60.6 |
| *R2R3-MYB* | F:GCGATCAAGAACCACTGGAACTCC | 24 | 60.5 |
|  | R:CGGCGTCTAGGCTGCTGATTTC | 22 | 61.3 |
| *GGPPS* | F: GCCATCATCAGTGGTGCTTCAGAG | 24 | 60.3 |
|  | R: CCAACAACGGTGCCGCCTTC | 20 | 59.8 |
| *DXS* | F: GATGGAGCCATGACAGCAGG | 20 | 60.8 |
|  | R: GCCCATCCACAGGTCCAATG | 20 | 61.4 |
